# Investigating the impact of the dispersion protocol on the physico-chemical identity and toxicity of nanomaterials: a review of the literature with focus on TiO_2_ particles

**DOI:** 10.1186/s12989-025-00627-8

**Published:** 2025-05-13

**Authors:** Andrew McCormack, Vicki Stone, James McQuat, Helinor Johnston

**Affiliations:** 1 Institute of Biological Chemistry, Biophysics and Bioengineering, School of Engineering and Physical Sciences, Heriot-Watt University, Edinburgh, UK; 2 Publishing Consultant, Winchester, UK

**Keywords:** Dispersion protocol, Agglomeration, Sonication, Dispersant media, Titanium dioxide, Reactive oxygen species, Cytotoxicity, Nanoparticle, Nanomaterial

## Abstract

Particles often require dispersion in aqueous media to allow assessment of their hazard profile. The approach used to disperse particles is not consistent in the published literature, with approaches including stirring, vortexing, shaking or sonication, and the use of biological or chemical stabilisers. Such variations in the dispersion protocol can influence the physico-chemical (PC) identity and toxicity of particles. To better understand the protocol variations and their impacts on human health, this work identified and critically reviewed publications with a specific focus on titanium dioxide (TiO_2_), which was dominated by nanomaterials (NMs). This review included consideration of both in vitro and in vivo studies, as well as other NMs to help address knowledge gaps and identify any lessons that can be learnt and applied to TiO_2_. Overall, the evidence gathered showed that variations in the dispersion protocol, specifically the method and parameters of sonication (e.g. power and duration), as well as the dispersion medium choice (and inclusion of biological and chemical stabilisers), were impactful on NM agglomerate size. There is no consensus as to whether a reduction or increase in NM agglomeration enhances or reduces NM toxicity with the outcome of the study dependent on the experimental design (e.g. PC properties of the NM being tested, test model used, time point, and concentrations/doses assessed). Whilst standard protocols for NM dispersion have been generated, they have not been widely adopted and there is unlikely to be one protocol that can be applied to all NMs and test models. Instead, more guidance is needed to inform the considerations that should guide preparation of NM suspensions for hazard testing. These include a recommendation that pilot studies are performed to identify the most suitable dispersion protocol before embarking on a toxicology study. Improved knowledge of the impact of dispersion protocols on PC identity and toxicity of TiO_2_ will assist in the interpretation of existing toxicology data and feed into the design of future studies which assess TiO_2_ toxicity.

**Supplementary Information:**

The online version contains supplementary material available at 10.1186/s12989-025-00627-8.

## Background

When investigating the toxicity of particles using in chemico, in vitro and in vivo approaches they typically require dispersion in biological media so that the test model (e.g. cells, animal) can be exposed effectively. The dispersion strategy is primarily employed to better reflect the environmentally relevant exposure, including break up of particle agglomerates.

However, the dispersion of particles in physiologically relevant solutions can promote transformations such as agglomeration, dissolution, or a change in surface properties. Such changes in the physico-chemical (PC) identity of particles can lead to issues with reproducibility and discrepancies between study findings [1]. This is because PC characteristics of particles are important in determining their behaviour in a suspension, such as the degree to which they aggregate or agglomerate [2]. Physical properties pertain to the structural features of the particles, for example particle shape and size distribution, while chemical properties are associated with the elemental or molecular composition of the particle, and thus include properties such as particle surface chemistry, surface charge, purity and hydrophilicity/hydrophobicity. When these PC properties are changed, the surface forces on the NM change, and thus interfere with adhesion, contact and deformation behaviour of the NM [3].

The impact of dispersion on PC properties has been most widely studied for nanomaterials (NMs), which have at least one dimension that is < 100 nm. For example, it has been demonstrated that larger agglomerates of NMs can exhibit different toxicity to well dispersed particulate suspensions [4–7]. The same substance can exist in various forms, exhibiting diversity in the PC properties such as size (nano, micro, macro), surface area, shape, surface properties etc. However, different forms of the same substance may not demonstrate dispersion propensity in different media, or the same hazard profile (e.g. spherical TiO_2_ might have a different hazard to TiO_2_ nanotubes). Additionally, a single substance can be sourced from a wide range of suppliers, for example NMs may be obtained from commercial chemical suppliers (e.g. Sigma Aldrich), NM producers, NM repositories or be synthesised in the laboratory by investigators.

Several approaches can be used to limit the agglomeration of NMs in biological media; each of which have their own advantages and limitations. Health Canada identified the uncertainty regarding relevance of the dispersion techniques used in existing in vitro and in vivo studies when assessing the impact of TiO_2_ on human health [8]. In particular the Health Canada report suggested that in the presence of light, TiO_2_ could undergo photocatalytic reactions. They suggested that if these reactions occur in the aqueous dispersion media the resultant hydroxyl radicals may lead to oxidative damage in biological test systems. Additionally, it was emphasised that when sonication was used to disperse TiO_2_ agglomerates, there may be a further generation of unwanted reactive radicals, which may contribute to TiO_2_ being genotoxic. However, a recent comprehensive review by Kirkland et al., (2022) [9] detailed the strength of the existing evidence to support the claim that TiO_2_ was genotoxic, and concluded that out of the 192 identified studies, only 34 met the reliability and quality criteria for evaluation of genotoxicity. Although the direct effects of the diverse range of dispersion protocols employed by TiO_2_ toxicity studies was not reported in detail, it was identified by Kirkland et al., (2022) [9] that the different dispersion strategies were significant contributors to differences in the PC properties of TiO_2_ between studies and resulted in difficulties when comparing each study’s findings.

This work will compare the different dispersion protocols used to prepare NM suspensions and identify the impact of these dispersions on the PC identity and the toxicity of TiO_2_, taking into account the strength of the evidence in published reports. We have also investigated whether there is sufficient evidence to support the claim that reactive radicals are produced during the dispersion of TiO_2_ suspensions, including whether sonication enhances radical production. It should also be noted that, whilst a focus will be placed on TiO_2_ particles, the majority of publications identified are focused on NMs. In addition, literature investigating other NMs is included to help address knowledge gaps and identify what lessons can be learnt and applied to TiO_2_. Finally, the results will be used to identify the possibility of standardising the dispersion protocol when testing the toxicological effects of TiO_2_.

## Methodology

### Identification of relevant literature

A review of the relevant literature was performed to assess the influence the dispersion protocol has on the PC identity and toxicity of particles. Identification of literature was achieved by searching PubMed, using keywords such as ‘particle size’, ‘agglomeration’, ‘dispersion protocol’, ‘sonication’, ‘nanoparticles OR nanomaterials OR particles’ ‘in vitro’ OR ‘in vivo’, ‘cytotoxicity’, ‘oxidative stress’, ‘genotoxicity’, ‘titanium dioxide’, ‘TiO2’. A search of the literature was conducted between Jan-Mar 2024. Relevant studies were identified to be published between 2007–2023. Additionally, any research publications which were referenced in these studies and deemed relevant to our review were included. Alongside academic research papers, reports produced by industry, government organisations and international regulatory bodies, which contained relevant content were also considered for inclusion. Following identification, the quality of the literature was assessed by evaluating the relevance of study endpoints, aspects of the methodology (e.g. controls, number of replicates), test substance characterisation, and plausibility of the results, against specific criteria as described by Klimisch et al., (1997) and Schneider et al., (2009) [10, 11].

### Assessing of dataset quality

This work implemented a reliability assessment tool, named the ToxRTool (Toxicological data Reliability Assessment Tool) which provides a guided assessment of the data to facilitate assignment of a Klimisch Score, either 1 (high quality), 2 or 3 (low quality). The score was verified by the reviewer and modified where sufficient reasoning was identified. Table 1 provides details on how the Klimisch Score is defined and can be interpretated by the reader [9–11]. Briefly, the ToxRTool assigns a ‘0’ or ‘1’ to a range of criteria, representing whether the reviewer believes there is evidence in the study to support that a single criterion has been met (‘yes’ reflected by ‘1’) or not (‘no’ reflected by ‘0’). These 1’s and 0’s are then tallied to provide an overall score, that can be translated into a Klimisch category. The ToxRTool lists 21 and 18 criteria for in vivo and in vitro studies, respectively. Of these criteria, several are considered to be critical to having a minimum degree of data reliability and as such are marked in red. In the case that one of the red criteria is unfulfilled, a Klimisch Score of 3 is assigned, irrespective of the total sum of the criterion [10]. This tool could not always be applied to the identified literature as not all studies consulted were in vitro or in vivo studies. For example, the ToxRTool was not used for studies whose hypotheses were tailored towards developing an understanding of the effects of the dispersion protocol on the PC characteristics of a particle, rather than on particle toxicity.

The ToxRTool was also used for assignment of a NanoScore [12]. This score (in the range of 0–10) was used to evaluate the degree of critical evaluation of the particle PC properties provided by each study, where a maximum score of 10 indicates robust and thorough characterisation. Again, the ToxRTool assigns a ‘1’ where appropriate characterisation was carried out and a ‘0’ if the parameter was not assessed. It should be noted that where certain PC properties were characterised by the particle supplier and not verified by the authors of the study, a ‘0’ was assigned [9]. In the case that the authors stated that a PC characteristic, e.g. particle size, was assessed in a previous study by the same research group, and the data could be verified, a score of ‘1’ was assigned [9]. A complete list of the PC properties included in the determination of the NanoScore is provided below.

- Agglomeration and/or aggregation.
- Chemical composition.
- Crystal structure/crystallinity.
- Particle size/particle distribution.
- Purity.
- Shape.
- Surface area.
- Surface charge.
- Surface chemistry (including composition and reactivity).
- Whether any characterisation was conducted in the relevant experimental media.

**Table 1:** Definition and explanation of Klimisch scores

Klimisch Score	In vivo criteria (out of 21)	In vitro criteria (out of 18)	Category Definition (as assigned by Klimisch et al., 1997)	Interpretation of Score
1	18–21	15–18	Reliable without restriction	The study follows generally accepted guidelines/standard procedures for carrying out tests and is both transparent and detailed in its reporting of the results. The data is thus considered reliable.
2	13–17	11–14	Reliable with restrictions	The study exhibits deviations from accepted guidelines/standard procedures for carrying out tests, however the test parameters are considered to be sufficient to accept the data and the methodologies are considered to be scientifically acceptable. The data is thus considered to be reliable, however the applicability of the study/relevance of the data to an intended aim is at the discretion of the reviewer.
3	< 13 or not all red criteria met.	< 11 or not all red criteria met.	Not reliable	The study reports a test procedure which may not be relevant/scientifically acceptable e.g. there is some interference between the test substance and the measuring system. *Or* the reporting of the test is not sufficient to convince the reviewer that the test procedure is acceptable. The data is thus generally considered not to be reliable, however the shortcomings of the study must be carefully considered, and the data may yet be deemed useful.

## Results

Publications were identified and reviewed based on whether they investigated the impact of variables of the dispersion protocol on the PC identity and toxicity of a substance. In total, 23 papers were identified which met this criterion. In all of these studies, the substance existed in a NM form, and for this reason the following text uses the term NMs rather than particles. Of the studies identified, 11 focussed on TiO_2_ while 12 papers assessed other substances, including silicon dioxide (SiO_2_), zirconium dioxide (ZrO_2_), ceric dioxide (CeO_2_), carbon nanotubes, aluminium oxide (Al_2_O_3_), nickel oxide (NiO), zinc oxide (ZnO), copper (Cu) nanoparticles, silver (Ag) nanoparticles, iron oxide (Fe_2_O_3_) and sepiolite NMs. Of the studies which investigated the impact of the dispersion protocol on the toxicity of a substance, 11 out of 12 performed in vitro assessments, with only 1 identified paper performing an in vivo hazard study. In addition, 11 studies were identified to focus solely on the impact of the dispersion protocol on the PC properties of a NM stock suspension. Interestingly, existing toxicology studies predominantly focussed on inhalation as the route of exposure with less emphasis placed on other exposure/target sites (e.g. intestine). This is due to the potential for repeated occupational exposure to dry powders, including TiO_2_.

We found that characterisation of the PC properties of particles was performed for two main purposes in the studies reviewed. Firstly, as the information provided by commercial suppliers is not always accurate, independent characterisation was commonly performed to confirm the PC properties of the test material. Furthermore, when working with lab synthesised materials there was a need to confirm their PC properties. Characterisation of particle PC properties is also performed to identify whether the PC properties of particles changed when they were dispersed in biological media, and typically a focus was placed on assessing particle agglomeration or aggregation as this is particularly important when determining what impact, the dispersion protocol has on the PC identity of a particle.

### Source of nanomaterials identified in publications

**Fig. 1:**
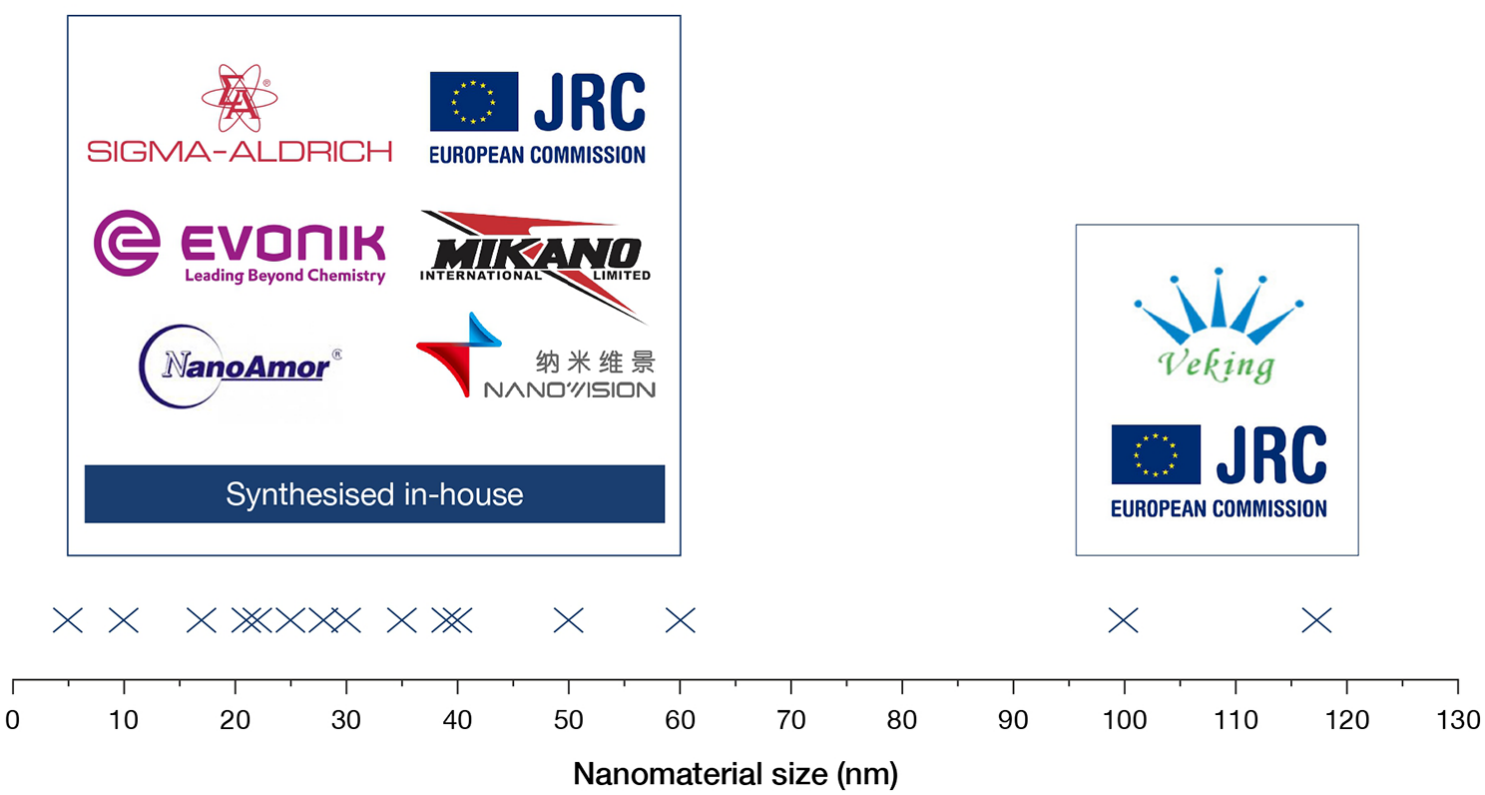
The range of TiO_2_ nanomaterial sizes and suppliers assessed in this report. These nanomaterials were used to assess what effects the dispersion protocol has on NM PC identity and toxicity

(Fig. 1) details the range of sources of TiO_2_ particles that were reported in the literature and states the diversity in size of these materials. Our observations aligned with those of others, in that a limitation for publications assessing NM toxicity is identifying whether the NM tested represents a form of the material that closely mimics what humans will be exposed to [8, 13]. For example, when the toxicity of ingested TiO_2_ has been investigated studies commonly neglect to test food-grade materials such as forms that are used as food additives (e.g. E171). E171 specifically, is comprised mainly of microparticles, with only around 25% of the TiO_2_ existing in the nanoscale [14].

The majority of studies we reviewed sourced NMs from commercial suppliers or the Joint Research Centre (JRC) NM repository, with few studies synthesising their NMs ‘in-house’. Materials such as those from the JRC repository have been used in international testing programmes (e.g. the Organisation for Economic Co-operation and Development (OECD) Working Party on Manufactured Nanomaterials testing programme) which have gathered vast amounts of hazard and safety data on a wide range of materials. The PC properties of the (as supplied) materials in such repositories have also been extensively investigated.

### Nanomaterial agglomeration and aggregation

**Fig. 2:**
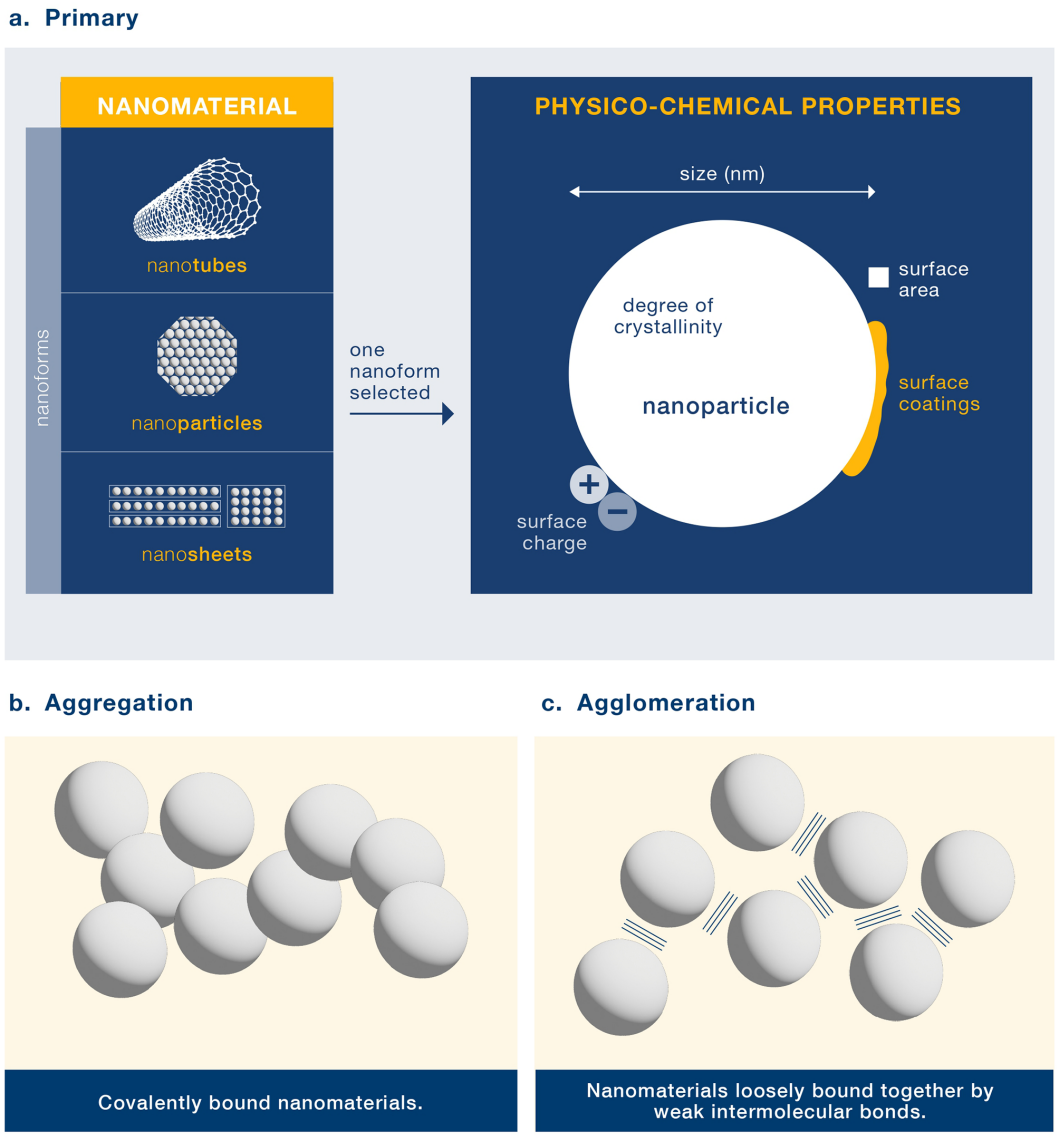
Representation of nanomaterials and their aggregates or agglomerates. (**a**) Primary NMs can exist in a number of forms (e.g. varying in shape, crystal form, size, surface area, charge and coating). These forms can exist as primary particle, aggregates or agglomerates. Whilst not represented in the figure, it is possible that aggregates of nanomaterial agglomerates exist in the suspension. (**b**) Aggregation of NMs via covalent bonds. (**c**) Agglomeration of NMs via weak intermolecular bonds

We found that the terms agglomeration and aggregation were often used interchangeably but these terms have distinct definitions. Weak interactions (e.g. Van der Waals forces) bind agglomerates of particles together, and the formation of such structures is reversible, whereas for aggregates, particles are more strongly bound together (e.g. covalently) (Fig. 2) [15, 16].

The influence that particle agglomeration has on the biological response has been investigated in vitro and in vivo, with inconsistent findings observed (i.e. there is evidence that agglomeration can enhance or reduce NM toxicity). Details from several studies summarising the impact of agglomeration of NMs on their toxicity to both in vitro and in vivo systems are presented in Additional File 1: Table S1. Evaluation of the evidence presented in these studies, including the reliability of the datasets and characterisation of the NM PC properties is also provided, via assigning of both a Klimisch- and Nano-Score. The evaluated studies were predominantly assigned a Klimisch Score of 3 suggesting that there could be concerns over the reliability and quality of data [9]. In these cases, the low Klimisch score generally reflected the use of a small number of replicates in the conducted toxicity assays, which detracted from the strength of the quantitative conclusions. Many studies were assigned a NanoScore of < 5 suggesting that a more comprehensive assessment of the PC properties of the NMs could have been performed. As studies were focused on analysing the effects of agglomerates on NM toxicity, they typically reported agglomeration size, particle size, shape, zeta potential and generally performed their analysis in relevant biological media.

Both in vivo (e.g. rat or mouse) and in vitro (cell based) studies have compared the toxicity of small or large agglomerates of NMs and have obtained inconsistent findings (Additional file 1: Fig S1). For example, Murugadoss et al., (2020) modified the pH of the dispersion medium to influence particle agglomeration and observed that larger agglomerates of TiO_2_ NMs elicited stronger toxic responses in vitro (inflammation, oxidative stress and DNA damage (THP-1 cells)) and in vivo (pulmonary inflammation). At pH 2, TiO_2_ NMs composed from 17 nm particles were well dispersed, and tended to aggregate when prepared at pH 7.5. The opposite was observed for 117 nm TiO_2_, where particles were less agglomerated when dispersed at pH 7.5 [16]. Overall, larger agglomerates of TiO_2_ NMs elicited a stronger toxic response across both in vivo and in vitro studies. However, on the cellular level, the stronger effects observed in macrophages were mostly attributed to the larger amounts of material deposited on the cells, rather than only the size of the larger agglomerates. Larger agglomerates resulted in higher doses on the cellular surface due to faster sedimentation, increasing the potential for cellular stress and toxicity. In the instance where the primary particle size was larger (117 nm compared to 17 nm) it was speculated that even smaller agglomerates were already of the optimal size for phagocytosis to be promoted [16]. Similarly, Noël et al., (2012) [17], demonstrated that larger agglomerates (> 100 nm) of TiO_2_ NMs induced a greater pulmonary inflammatory response than smaller agglomerates (< 100 nm) in the rat lung following inhalation. While the in vitro studies of Hu et al., (2019) [18] and Sharma et al., (2014) [19] show that smaller NM agglomerates elicit greater toxicity than larger agglomerates, with Sharma et al., (2014) [19] showing evidence that smaller agglomerates of carboxylated iron(III) oxide (Fe_2_O_3_) induced greater cell death and increased oxidative stress when compared with large agglomerates. Interestingly, in the latter case, if we were to compare Fe_2_O_3_ and TiO_2_ NMs, these materials exhibit differing PC characteristics such as composition, size and solubility as well as differing stable oxidation states at physiological conditions. While Fe_2_O_3_ and TiO_2_ show a very similar low solubility, Fe_2_O_3_ is generally considered slightly more soluble than TiO_2_, where Fe ^2+^ ions readily dissociate in water and can catalyse hydrogen peroxide (H_2_O_2_) oxidation (a process known as Fenton’s reaction [20]), to produce toxic reactive oxygen species (ROS). Thus, in the case of Fe_2_O_3_ NMs, which can undergo a redox-reaction to release divalent cations, surface area may be a more pronounced contributor to toxicity than for an even lower solubility material such as TiO_2_. Finally, there are examples of studies (e.g. Gosens et al., 2010; Peng et al., 2014; Sharma et al., 2014 [19, 21, 22]) which report that small and large agglomerates of NMs elicited comparable toxicity.

The conclusions are likely to vary between studies due to; the wide range of test models that have been used across different studies (e.g. species (and route of administration), cell type), differences in the doses/concentrations of NMs that were administered and time points assessed as well as variation with respect to the PC properties of the NMs (e.g. composition, primary particle size, agglomerate size, solubility) that were selected for investigation and the evaluation of different markers of toxicity. These findings highlight the need for more investigations into the influence that particle agglomeration has on toxicity, and in particular the role that primary particle size has on the toxicity of agglomerates. While many of the studies, specifically reviewed in this manuscript, put emphasis on studying the relationship between PC properties of NMs and markers of toxicity, there were limited studies which investigated changes in cellular uptake of NMs. The studies reviewed here, often only go as far to infer cellular uptake of NMs by reporting for example, the percentage of cells undergoing apoptosis and necrosis [23]. Pradhan et al., (2016) [24] are one of very few studies, which analysed the released metal in solution from various NMs using atomic absorption spectroscopy as a method to study, more closely, changes in cellular uptake of a NM over time. Out with the studies reviewed here, there are examples in the literature which review how the degree of NM agglomeration influences how cells internalise them [25–27]. For example, Halamoda‑Kenzaoui et al., (2017) [26], showed that smaller agglomerates of Ag NMs were more readily taken up by the cells. It therefore could be recommended that more investigations should be conducted into the effect changes in agglomeration/aggregation influence cellular uptake, for example by evaluating the concentration of specific analytes internalised by a cell in response to exposure to a NM by e.g. Inductively Coupled Plasma Mass Spectrometry (ICP-MS).

Furthermore, particularly for in vitro testing, reporting on the impact of the homogenous phase suspending NMs is not well-studied. For example, media may promote agglomeration (via ionic interactions), encouraging denser agglomerates, promoting faster sedimentation and affecting the amount of material deposited on cells.

### Strategies to reduce nanomaterial agglomeration

The tendency of particles to agglomerate in aqueous media has meant that different strategies have been used to improve the dispersibility and stability of particle suspensions, such as sonication (bath or probe), stirring, shaking, vortexing, and the inclusion of solvents (e.g. dimethyl sulfoxide (DMSO), ethanol, tetrahydrofuran (THF)), surfactants (e.g. Tween 80) and biological dispersants/stabilisers e.g. proteins (e.g. albumin, serum, and bronchoalveolar lavage fluid) (reviewed by Hartmann et al., 2015 [28]). In this work, the term dispersibility refers to the tendency for aggregation/agglomeration of NMs to occur in solution (with limited/no agglomeration observed in well-dispersed suspensions), while stability describes the influence that agglomeration has on the subsequent settling of NMs over time.

**Fig. 3:**
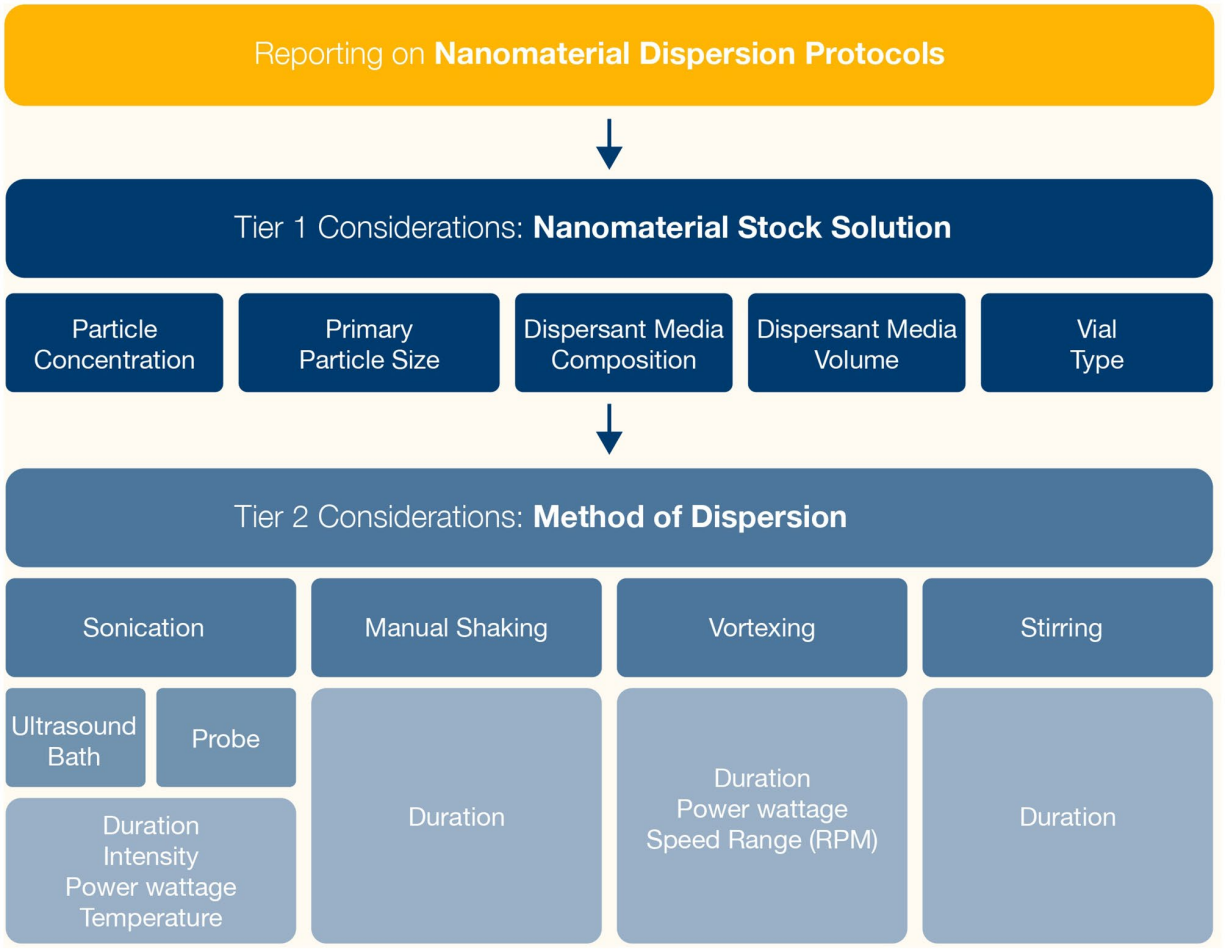
Schematic overview representing the different steps involved in dispersing NM suspensions. In the first instance, a stock suspension of NMs is prepared. However, the approach used to prepare the stock suspension is not consistent. Once the stock suspension is prepared it may be used immediately or one of several approaches may be used to improve the dispersion of the suspension. Following stock suspension preparation, typically one method of dispersion is applied (e.g. sonication or use of a dispersant) but some studies have used a combination of approaches to prepare NM suspensions (e.g. sonication and use of a dispersant)

The literature search provided further evidence that the dispersion protocol can influence the PC identity of particles (e.g. size, agglomeration, charge, surface chemistry, morphology, solubility) and therefore their fate and toxicity [4, 5, 24, 29–33]. Ideally, dispersion strategies should be relevant to the environmental or physiological conditions of the exposure route of the particle being studied, striking a delicate balance between preparing the ‘best possible’ dispersion of a particle suspension (that minimises agglomeration) and mimicking the in vivo situation [34]. Additionally, the dispersion strategy should take into account the strength of the primary particle binding within agglomerates and the viscosity of the homogenous phase (i.e. the dispersant media) [35]. Typically, for aqueous dispersant media, which exhibits relatively low viscosity values, sonication methods are more frequently reported in the literature than manual shaking/stirring and vortexing [7, 16, 28, 35, 36]. Often, a combination of approaches, including sonication methods and biological dispersants are used to prepare ‘well dispersed’ particle suspensions (Fig. 3), which strike the balance of achieving a suspension of particles that is less likely to agglomerate against introducing potentially toxic substances, generating ROS or damaging the particles or biological dispersants/stabilisers via use of sonication [28].

Such protocols have been considered appropriate to [5, 28]:

- Enhance the stability of particle dispersions,
- Ensure reproducible dispersions are generated,
- Improve the delivery of particles to the test model to better mimic the environmental or physiological conditions experienced by the particle,
- Minimize variation between test systems, labs and particles,
- Identify which particle PC properties confer toxicity,
- Prevent the domination of physical effects associated with particle agglomeration when assessing a hazard.

The relevancy of using these approaches to ‘real life’ human or environmental exposures have however also been questioned [5, 28].

Several attempts to generate useful dispersion protocols have been published (e.g. Jensen et al., 2011; Kaur et al., 2017; Prospect, 2010 reviewed by Hartmann et al., 2015; [28, 37, 38]). Since a combination of approaches have been used, it is difficult to dissect the role that each step of the dispersion protocol has on the PC identity and toxicity of the particle. It is therefore important to distinguish between the inherent properties of a particle that are driving the toxic response and the ability of the particle dispersion method to cause artifacts as this can compromise the conclusions that are made [7].

NM stock suspensions are commonly prepared prior to carrying out toxicological studies. Many factors relating to the preparation of the NM stock suspension play a role in the extent of the agglomeration status of the NMs [9, 28], including primary particle size, particle concentration, volume of the stock suspension and the vial type (Fig. 3), as well as the time elapsed between dispersion and application. For instance, high stock suspension concentrations promote a higher degree of agglomeration, initially, due to an increased frequency of collisions between particles [24]. It has also been discussed that the smaller the primary particle size, the higher the forces of attraction per unit mass are [33], inhibiting easy de-agglomeration of the NMs.

Details regarding the preparation of the stock suspension are frequently neither discussed nor reported in the literature. Thus, it should be considered that reporting of the dispersion protocol should include information not only on the method of dispersion but also details on the preparation of the stock suspension (Fig. 3).

These stock suspensions can then either be diluted, for instance to achieve a desired concentration or replenish culture media and nutrients, or they are used directly in the hazard study. It is advised that the stock suspension is not stored prior to use, although on some occasions NM suspensions have been stored in the fridge or freezer to aid long-term stability of dispersion [40–42]. In the following sections of this article, we will explore the impact of different dispersing methodologies on the NM PC identity and the toxicity of the NM.

#### Dispersant media and inclusion of stabilisers

In a particle suspension the choice of dispersant media and the possible inclusion of stabilisers (biological or chemical) is critical to achieving a ‘well dispersed’ suspension of particles, due to their interaction with the particle surface. In the presence of complex biological fluids, the spontaneous adsorption of proteins (such as serums (e.g. FBS/FCS)), lipids or other biological moieties onto the particle surface can occur, altering the dispersibility and stability of the NM [43]. The adsorption of these biologically active molecules can promote the formation of a ‘protein corona’ on the particle surface, affecting the agglomeration status of the particle, the interaction of the particle with cells and potentially masking the reactivity of the particle [43, 44]. The structure and function of the media components is thus important in influencing both the dispersion of the particle and the resulting toxicity [43].

The inclusion of ‘natural’ physiologically relevant stabilisers can better mimic human or environmental exposure as particles would be expected to interact with these molecules following exposure to humans or release into the environment. Ideally, the use of such stabilisers should also be at biologically relevant concentrations (although this is not always the case). In vivo (rodent) studies typically disperse NMs in saline in the presence or absence of physiologically relevant (e.g. surfactants, proteins) or chemical stabilisers. In vitro experiments with mammalian cells typically (but not always) prepare NM suspensions in cell culture medium that contains serum.

It is established that serum proteins can reduce the agglomeration of NMs [30, 45, 46]; it is anticipated that the mechanism involves the NMs becoming coated with serum proteins to improve their repulsion and therefore improve dispersion. However, there are discrepancies regarding what impact the inclusion of serum has on NM toxicity. Magdolenova et al., (2012) [47] demonstrated that TiO_2_ NMs prepared in the presence of serum (20% FBS) were less agglomerated and less toxic than the TiO_2_ suspensions prepared in the absence of serum which contained larger agglomerates. Similarly, Drescher et al., (2011) [48] investigated what impact the FCS concentration (0%, 1%, 5%, 10%) had on the toxicity of amorphous silica to fibroblasts in vitro and observed that NM mediated cytotoxicity increased as the concentration of FCS decreased which could be related to their agglomeration or the availability of surface chemical groups. Furthermore, Murdock et al., (2008) [30] demonstrated that the toxicity of NMs (e.g. silver (Ag), copper (Cu)) was enhanced in the absence of serum. In contrast Cronholm et al., (2011) [36] observed that the toxicity of Cu NM in vitro (A549 lung epithelial cells) was enhanced when dispersed in medium containing serum for some (but not all) endpoints. The presence of serum can also enhance the dissolution of metal/metal oxide NMs to influence their toxicity [36], and potentially provide binding sites for such cations.

In addition to affecting the agglomeration status of particles, a lack of serum will have other implications for cell function (e.g. as serum contains nutrients and growth factors required for cell growth) and so it can be challenging to distinguish what effects are attributed to particle agglomeration. For this reason, it is essential to include appropriate controls in hazard studies (e.g. cells should be exposed to media lacking serum in the absence of NMs).

It is not only the inclusion of serum that can influence the biological response, but the level of serum that is present and the source of serum (e.g. animal vs. human) [31, 45]. Serum is a complex mixture of proteins of which albumin is the most abundant, and thus albumin has been included in NM dispersions in order to mimic the proteins NMs interact with in a physiological environment. For example, bovine serum albumin (BSA) or human serum albumin (HASA) have been demonstrated to reduce the agglomeration of NMs, however the affinity of albumin proteins for different NM types varies and binding of albumin to the NM surface is influenced by the pH and ionic strength of the media [31, 32, 49]. Interestingly, FBS generates better dispersions of TiO_2_ NMs than albumin, which suggests that there are different components in serum which can improve the stability of NM dispersions [49].

The use of some dispersants within NM suspensions used for in vitro studies can increase their relevance to the in vivo situation that they mimic [34]. For example, bronchoalveolar lavage fluid (BALF) has been used to prepare NM suspensions for in vivo and in vitro studies which assess the pulmonary toxicity (e.g. Sager & Castranova, 2009 [5]). The use of BALF improved the dispersibility of silica NMs but did not affect their bioactivity in vitro and in vivo when investigating pulmonary toxicity [5]. However, whilst BALF may be a good vehicle for dispersing NMs there are several limitations associated with its use, such as, the ethical implications of obtaining BALF samples from species (e.g. mouse, rat, human) relevant to the test model, the intra and inter-laboratory variability in BALF samples and the time required to generate BALF samples [50]. Therefore, the inclusion of physiologically relevant proteins and surfactants (e.g. 1, 2-dipalmitoyl-sn-glycero-3-phosphocholine (DPPC)) in NM suspensions has been explored as an alternative to BALF. For example, Porter et al., (2008) [50] demonstrated that the inclusion of pulmonary surfactants reduced the tendency of NMs to agglomerate but did not enhance their toxicity in vitro or *in vivo.* Brown et al., (2014) [51], compared the toxicity of NMs that were dispersed in suspensions containing serum, lung lining fluid or albumin, and found that the toxicity of NMs was enhanced in the presence of serum when macrophages were used as the in vitro test model. Therefore, existing evidence suggests that the choice of biological dispersant can influence the outcome of toxicity studies. Sauer et al., (2015) [34] compared the dispersing properties of BSA and (commercially available) porcine lung surfactant and it was demonstrated that BSA was most effective at dispersing NMs. Interestingly, it was observed that different NMs do not respond in the same way to different dispersants. Thus, the approach used for dispersion may need to be tailored to the NM under investigation.

For ecotoxicology studies natural organic matter (NOM) can be included in NM suspensions to better reflect real-life exposures. It is thought that NOM adsorbs onto the NM surface following their release into the environment to influence their fate and ecotoxicity, and it has been commonly observed that organic matter reduces the toxicity of NMs [52]. For example, Little et al., (2021) [53] included humic acid in NM (TiO_2_ and Ag) suspensions to improve environmental relevance when investigating acute aquatic toxicity (using as a test model, Lumbriculus variegatus), and observed that it could mitigate against NM mediated toxicity.

As an alternative to biological molecules, chemicals (e.g. solvents) can be included in particle suspensions to improve their dispersion and stability. However, it is imperative to confirm that the inclusion of such chemicals does not influence the toxic response that is observed. For example, it has been proposed that tetrahydrofuran (THF) enhances the toxicity of fullerene (C60) [54]. Several NM protocols recommend pre-wetting (hydrophobic) NMs with ethanol (or other solvents) to improve their dispersion (e.g. Jensen et al., 2011 [37]), through lowering of the surface tension of the dispersant [28]. For example, in the NANOGENOTOX dispersion protocol [37], a 0.5 vol % ethanol solution is used for prewetting of NMs to aid dispersibility. While the concentration of ethanol is low, this still may contribute to any observed toxicity when exposing cells/animals to NM dispersions prepared using this methodology. One example of research conducted on HepG3 human hepatoma cells indicated small ethanol concentrations (1 mmol, approximately 0.0046% v/v) inhibited cell proliferation and increased apoptosis compared to normal rat hepatocytes [55]. While these findings suggest that small concentrations of ethanol have adverse effects, these may be dependent on the cell line. For example, a study assessing the cytotoxic effects of ethanol on RAW 264.7 (macrophage), MCF-7 (breast cancer) and HUVEC cell lines showed only a small decrease in cell viability as ethanol concentration was increased from 0 (100%) to 0.5 v/v % (80–90%) [56]. Thus, it is essential that an appropriate vehicle control is included in each experiment to confirm that the stabiliser (chemical or biological) is not contributing to the toxicity observed. It is also important to assess the impact of such substances on the NM so that it remains representative of real-life exposures.

Studies which have assessed whether biological or chemical stabilisers influence the PC identity or toxicity of NMs are summarised in (Additional file 2: Table S2). These findings are consistent in reporting that agglomeration is promoted in cell culture media lacking serum, compared to water [30, 42, 48], with Schulze et al., (2008) [46] specifically identifying that the addition of Cl^−^ ions in the media will increase the agglomerate size. Zeta potential (which describes the potential difference between the NM surface and the surrounding liquid phase) of NMs was also shown to reliably tend towards smaller values when NMs were dispersed in culture media compared to water, conferring particle instability and their tendency to agglomerate [31, 57]. Furthermore, the addition of serum (FBS/FCS/BSA) to NM stock suspensions prepared in either water or cell culture media reduced agglomeration/aggregation [30, 31, 46, 57]. However, it should be noted that in some instances the addition of serum had the opposite effect on NM agglomeration, where NM agglomerate size increased [48, 58]. In regard to the toxicity of NM dispersions, there is evidence that serum-containing media may improve [48] or have no effect [30] on toxicity in vitro, compared to serum-free NM dispersions.

#### Sonication

Sonication is one of the most widely used approaches to improve the dispersion of particles and reduce the likelihood of particle agglomeration. This method of dispersion acts to overcome the inter-particle adhesion forces, primarily via the generation of a strong force created from a phenomenon known as ‘acoustic cavitation’. Under the action of ultrasound, mechanical vibrations travelling through the liquid phase in a particle stock suspension, initiate the growth and collapse of microbubbles, which upon collapse, generate several physical effects including, shock waves, turbulence and shear forces [59]. Collapse of these bubbles takes place in microseconds, and is considered an adiabatic process, meaning the high temperatures and pressures that are reached inside the bubble due to gas compression, are transferred to the surroundings, without loss of heat or energy [60].

While the liquid perturbations are beneficial to maintaining a good dispersion and preventing sedimentation of particles, both the generated high temperatures (which can be in the order of hundreds of degrees Celsius) and high strain forces are capable of breaking bonds inside a molecule [60]. As mentioned previously (Sect. 2.3.1), such effects can degrade and/or deteriorate the dispersant media as well as damage the NM (i.e. structural integrity, surface chemistry, surface charge). Due to these potential detrimental impacts on media components and dispersants, some publications recommend the sonication process in water, and then dilute in the desired biological medium [7, 31].

Sonication may also have an impact on the surface coatings of particles, where the deterioration of coatings could release toxic by-products into particle suspensions or completely change the PC identity of a particle [8, 9]. This is of particular concern when assessing the hazard profile of TiO_2_ forms, where often coatings such as magnesia, silica, alumina and zirconia are used to minimise the photocatalytic activity of TiO_2_ [61]. However, there are a lack of experimental studies which have assessed the impact of sonication on the integrity of NM surface coatings.

There is a lot of variation with respect to the type of sonication that is used (probe, bath or cup horn), the duration, the power, the choice of media, the volume, and the vial type to prepare NM suspensions. Evidence indicates that these factors can influence the characteristics of the dispersion following sonication and their subsequent toxicity [38]. In ultrasonic baths, acoustic cavitation occurs uncontrollably and is heterogeneously distributed through the liquid phase [62], while for ultrasonic probe devices, the ultrasonic effects are highly controllable and therefore more reproducible, permitting a higher intensity of sonication that is coupled with a further increase in heat imparted on the NM stock suspension. This in turn has implications such as, reducing the sample volume (which may impact on concentration of the NM) and promoting degradation of NMs or media components. Thus, use of an ice bath during probe sonication is often recommended (e.g. Betts et al., 2013 [63]).

For probe sonication, the metal tip can erode over time, and components (e.g. titanium) can be released into the NM suspension during long sonication times (≥ 30 min, in the instance the tip is new and not eroded) and such contamination could influence the toxicity of the suspension [7, 40, 63, 64]. Furthermore, for probe sonication, as the tip is in direct contact with the NM suspension being sonicated, the tip could become contaminated with the NM contained in the stock suspension [38], however the implications of this on toxicity has not been investigated.

A summary of the identified literature on the influence of sonication on NM PC identity and toxicity is provided in (Additional file: Table S3), with a corresponding Klimisch Score and NanoScore provided for each study, to inform the reader on the reliability of the datasets. These findings indicate the wide diversity of sonication protocols exploited, while highlighting how infrequently the impact of the sonication protocol on the agglomeration state *AND* their resultant toxicity is investigated. Instead, studies typically focus on either a change to NM PC identity *OR* the biological response to NM exposure, but not commonly both. Interestingly, probe sonication appears to be the preferred sonication method to generate ‘better dispersed’ NM suspensions, however variation of the sonication methodology such as the sonication time greatly differ, ranging from as short as 60 s [23] to longer times of 16 min [65]. In conjunction with a wide range of ambiguities embedded within the cited sonication protocols, we find there to be a consistent lack of reporting on specific variables within each protocol. For example, cooling systems are mostly used alongside probe sonicators, yet the sonication temperature is never specified [16, 23, 36, 66–68], preventing any assessment of how the temperature may damage/degrade the NM or biological components in the stock suspension.

Reporting on the sonicator settings is generally not standardised, with researchers frequently opting to report solely, either sonication time, power of sonication, or the ‘expected’ wave amplitude (%) and frequency (Hz) of the sonicator. This leads to challenges in defining the amount of delivered energy dose to the NM suspension per unit volume [7, 28]. This delivered energy dose is the direct consequence of an energy transformation occurring within the sonicator device, whereby the input electrical energy is converted to acoustic energy. The inertial forces required to overcome the attractive Van der Waals forces between NMs, is thus the result of the acoustic energy being delivered to the NM suspension [28]. Evidence shows that agglomeration is typically reduced with increasing sonication time and power, due to a higher delivered energy dose (e.g. Bihari et al., 2008; Pradhan et al., 2016; Wu et al., 2014 [24, 31, 40]), indicating that the delivered energy dose equates to the deagglomeration efficiency of the sonicator. The effects of applying this energy dose via continuous or discontinuous sonication, on NM agglomerate size and size distribution, has also been studied [66, 69]. These variations in the delivery of acoustic vibrations have been achieved either by adjustments of the ultrasound vibration pulse to 50% and 100% for discontinuous or continuous pulses, respectively [69], or through interjecting the sonication process with pauses for vortexing of the suspension [66].

These studies also highlight that there is some ambiguity to the definition of both ‘continuous’ and ‘discontinuous’ sonication, with the observed effects on NM agglomeration being quite different. Tajik et al., (2012) [69], show that continuous pulses (i.e. 100%) result in a higher incidence of deagglomeration and smaller, more uniform particles compared to discontinuous pulses. In contrast, Cohen et al., (2018) observed that discontinuous sonication (i.e. intervals of sonication interspersed with vortexing) is preferential for breaking up large agglomerates, and that this was interestingly only effective for rapidly agglomerating and settling NMs (Ag15% /SiO_2_, Ag and CeO_2_) compared to a slow agglomerating and settling NM (Fe_2_O_3_).

The findings from the studies presented (Additional file: Table S3), clearly indicate that sonication reduces NM agglomeration. It appears that probe sonication results in smaller agglomerates compared to bath sonication [24]. Similarly, Nickel et al., (2014) [70] demonstrated that TiO_2_ particle agglomerates were larger, and suspensions were less stable when bath sonication was used rather than probe. More studies would be useful to make a direct comparison between sonication methods.

Other than the effect of sonication on NM agglomerate size, the impact that sonication has on zeta potential and the release of metal ions (solubility) were also investigated in the literature. Pradhan et al., (2016) [24] demonstrate that prolonged probe sonication (15 min) results in no change to the zeta potential, while, Cronholm et al., (2011) [36] found that bath sonication increased the metal release from Cu agglomerates compared to non-sonicated samples. Again, there is not enough evidence to justify the validity of these findings, or whether these trends apply across a diverse range of NM substances.

In an attempt to establish better harmonisation to sonication protocols, several projects including NANOGENOTOX [37] and the European Commission Framework Programme (FP7)-funded Risk Assessment of Engineered Nanoparticles (ENPRA) project have proposed strategies to control the dose of delivered of energy to NM suspensions. These works discuss the implementation of developing a sonicator calibration method [37] or adopting the use of the same brand of sonicator across all research facilities to aid investigations into the acoustic energy dissipated by sonicators. However, these strategies have also come under some scrutiny [28], where for instance, the calibration method used in the NANOGENOTOX protocol [37] measures the consumed energy at the wall-fixture, which may not be representative of the electrical to acoustic energy transformation occurring within the sonicator itself, and thus makes calculating of the actual energy delivered to the sample challenging. The delivered dispersion energy was also alluded to in a recent report on the Guidance on the Safety Assessment of Nanomaterials in Cosmetics [71], where it was stated that typical probe sonication dispersion conditions involve applying energies between 600 J/mL and 2,500 J/mL relative to the sample volume.

The impact of sonication of a NM suspension to toxicity is both complex and difficult to interpret, due to the agglomerate sizes of NMs posing differing effects on acute and chronic toxicity depending on the experiment methodologies and the *in vitro / in vivo* model system used (Sect. 2.2). For example, there is evidence to suggest that cell viability is negatively affected by the sonication of NM suspensions, where Cronholm et al., (2011) [36] and Dai et al., (2019) [72], show a decrease in viability for lung (A549 cells) and macrophage (RAW 264.7) cell lines, respectively, when bath sonication was used to prepare NM suspensions. In contrast, Bettencourt et al., (2020) [65] and Brooks et al., (2022) [73] observe that toxicity is not enhanced by sonication, detailing that no effect on cell viability for epithelial (U2OS)/ fibroblast (RG37) and colon cancer (Caco-2) cell lines, respectively. Furthermore, NM aggregate/agglomerate size is shown to have differing effects on the toxicity to cells. Murugadoss et al., (2020) [16] found that for larger aggregates of 17 nm TiO_2_ particles, there was an increased production of proinflammatory cytokines (for HBE, THP-1 and Caco-2 cells) compared to smaller aggregates, while Hamzeh & Sunahara, (2013) [23] demonstrate that smaller aggregates of TiO_2_ particles have a greater adverse genotoxic effect on V79 cells. Subsequently, there is no conclusive evidence on the extent that sonication will affect toxicity or whether probe or bath sonication enhances/reduces toxicity the most.

In this review, experimental design of the toxicity tests was found to be a fundamental concern affecting the reliability of the studies presented in the literature, and the subsequent assignment of a low Klimisch Score. For example, while most in vitro tests reported their negative controls, the reporting of positive controls was uncommon, preventing reliable confirmation that any observed toxicity effects were the result of the NM and not an external factor. Typically, cell viability assays, such as MTT assays, exhibited no positive controls, however, assays focused on DNA damage (e.g. Comet assay) or cytokine production assays (e.g. ELISA), were more frequent in reporting of all their controls. The validity of studies was also limited by the use of a small number of repeats or small sample sizes. The probability that a significant effect was detected or that differences existed between two or more groups was thus compromised and limited the strength of the quantitative data.

The OECD state that particle size has a larger effect on particle settling than density and agglomeration leads to faster particle sedimentation [74], however there are few studies that review the relationship between particle settling, agglomeration and cellular dose. Studies such as Cohen et al., (2014) [75] and Sharma et al., (2014) [19] were considered how agglomeration can affect the delivered cellular dose, where Sharma et al., (2014) [19] specifically discuss that agglomeration could amplify the toxicity at lower NM concentrations, altering the way the dose-response profile is interpreted. Other factors which may affect the effective dose include the effective density [76], however the studies identified in this review did not evaluate this parameter. Further investigations into the relationship between effective density and cellular dose may be required to navigate how such measurements can be included during development of dispersant protocols.

### Reactive oxygen species generation in nanomaterial suspensions

The imbalance between the production of ROS and antioxidants in a biological tissue is known to have implications to multiple cellular organelles, disrupt normal cell functions and physiology and serve in the pathogenesis of a variety of diseases (Auten & Davis, 2009 [77]). More specifically, when the production of ROS overwhelms antioxidants (which are responsible for scavenging ROS), oxidative stress is activated. It is established that NMs such as TiO_2_ can elicit toxicity by promoting the formation of ROS in cells. For example Armand et al., (2016) [78], show that ROS levels in A549 lung epithelial cells exposed to 50 µg/mL TiO_2_ NMs were significantly higher than in cells exposed to 5 and 10 µg/mL TiO_2_ NMs. Of concern is that the sonication process could enhance the toxicity of NMs by promoting ROS production [6, 28, 79]. It was shown by Proquin et al., (2017) [79] that the food additive E171 when dispersed in Hank’s balanced salt solution (HBSS), generated significant levels of ROS following sonication (30 min at 40 kHz). More specifically, in an acellular environment, ROS production was observed for E171 as well as TiO_2_ nano- and macro-particles, for suspensions dispersed using bath sonication. Interestingly, ROS production was subdued when the dispersant medium contained 0.5% BSA, which was believed to be attributed to the formation of a protein corona on the NM surface that scavenged ROS [79]. In a cellular environment, only macroparticles of TiO_2_ were found capable of inducing ROS production [79]. No other studies were identified that investigated the impact that sonication had on acellular or cellular ROS levels in NM suspensions.

There is evidence in the literature that sonicated water generates OH and H radicals [80, 81] (through the process of acoustic cavitation) where sonication power and duration show a linear increase in radical formation, however these radicals have a very short lifetime [81]. Due to their high reactivity, the determination accuracy of ROS lifetime is challenging, however, Rubio & Cerón, (2021) [82] provide an approximate half-life of several ROS species at 37 °C (Table 2). It has therefore been debated in the literature that sonication of NM suspensions could promote ROS production which could influence their toxic potency. However, although generally not reported, the time from sonication to exposure of the test model (e.g. the addition of the NM suspension post sonication to cells) is very likely to be greater than several seconds, so it is unlikely that cells will be exposed to a large concentration of sonication-derived, active radicals. However, no experimental studies have investigated acellular ROS production in NM suspensions over time, following sonication.

**Table 2:** Approximate half-lives of selected ROS (reproduced from Rubio & Cerón, (2021) [82])

Molecular formula of free radical	Half-life, s (at 37 °C)
O_2_^−^	10^− 6^
.OH	10^− 9^
ROO	7
RO	10^− 6^

It has additionally been speculated that probe sonication could be a greater contributor to ROS production due to the higher intensity of acoustic vibrations delivered to NM suspensions compared to bath sonication [7], but this has yet to be confirmed experimentally. Further speculations include that NMs present in the sonicated suspension will be exposed to radicals (e.g. ROS) which could change their surface chemistry (e.g. via oxidative transformations) and can promote the leaching of ionic and soluble species [7], but no evidence in the literature was identified to support this hypothesis.

While it has been shown that serums in a dispersant medium may act to scavenge ROS radicals, there is also debate suggesting that sonication of serums may result in oxidative damage to the proteins, with ROS oxidising residue amino acids or even cleaving peptide bonds [83]. This could impact on the ability of the proteins to act as stabilisers or could contribute to the toxicity of the NMs due to a depletion of nutrients from the media. Therefore, until further investigations into ROS production for sonicated NM suspensions is conducted, it is recommended that either NM suspensions should be sonicated in aqueous mediums deprived of any biological components or that an appropriate control is included in all experiments where the media used to prepare NM suspensions is sonicated in the absence of NMs and exposed to the test model. ROS levels in the suspensions (in the presence and absence of NMs) should also be assessed immediately after they are prepared and over time (e.g. using the DCFH assay).

Health Canada place emphasis in their report [8] that TiO_2_ NMs exposed to light can catalyse the oxidation of hydroxide in water to produce ROS, where photocatalytic activity depends on the crystal structure of TiO_2_ i.e. whether it is anatase or rutile. However, to our knowledge there are no studies in the literature which assess how the dispersion protocol effects ROS generation via photocatalysis of TiO_2_. Due to the potential that TiO_2_ NM suspensions exposed to light may exhibit increased levels of ROS, we would thus recommend that suspensions should be prepared in the dark.

### Impact of storage conditions on nanomaterial physico-chemical identity and toxicity

The storage of particle stock suspensions is rarely discussed or reported in the literature, with very few studies providing details of the residence time of particles in the dispersant media prior to testing. It is often assumed that particle suspensions, following dispersion, are used *instantaneously*, however, there are studies which suggest that this is not the case. For example, Gutierrez et al., (2015) [84] state that “nanoparticles were stored in the dark and experimentations were initiated within 30 min of sonication”. Additionally, Magdolenova et al., (2012) [47], state that occasionally following sonication their stock solutions were “…stored at -20°C…thawed” then vortexed and sonicated again prior to testing. The storage temperature is also infrequently reported, with some studies storing stock suspensions at – 20–4 °C then diluting these suspensions when required [40, 41].

The effect of storage time and temperature of NMs in dispersant media on both NM PC identity and toxicity is inconclusive (Additional file: Table S4). It was found that these variables do impact NM PC identity and toxicity, but the extent of this impact and whether toxicity is enhanced or reduced is ambiguous. Murdock et al., (2008) [30] observed that the morphology of Cu particles, particle size and zeta potential changed over time (up to 34 days following preparation following storage at 4 °C) following the initial dispersion stock suspensions of particles. Interestingly, Bihari et al., (2008) [31] demonstrated that the stability of TiO_2_ NMs decreased over time (1 week) when dispersed in PBS (as indicated by an increase in particle size), but that dispersions remained stable when albumin was included. While, Kittler et al., (2010) [85] observed that the toxicity of Ag NMs increased during storage due to their dissolution, and that this was influenced by storage time (3 days, 1 month, 6 months). Thus, it is recommended that the storage time and temperature are reported.

### The physiological relevance of dispersion protocols

Physiological relevance relates to shifting the exposure protocol closer to that experienced in real exposure scenarios. For instance, for the lung, the use of submerged cell cultures can be exchanged for air liquid interface (ALI) in vitro systems to better reflect exposure in vivo. Interestingly, the pulmonary toxicity of NMs can be enhanced (e.g. Holder et al., 2008; Klein et al., 2013 [86, 87]), reduced (e.g. Bessa et al., 2021; Lenz et al., 2013; Lovén et al., 2021 [88–90]) or remain the same (e.g. Medina-Reyes et al., 2020 [91]) when they are applied in a suspension compared to when they are aerosolised. Therefore, the relevance of such models and exposure formats is still open to debate.

In addition, NMs have been subjected to digestion in acidic media and then neutralised to represent their transit through different compartments of the gastrointestinal tract (GIT). For example, Gerloff et al., (2013) demonstrated that simulated digestion did not impact on the solubility of ZnO NMs but that the surface reactivity (measured by electron paramagnetic resonance, EPR) of ZnO and SiO_2_ NMs was reduced after simulated digestion, however, no impact on cytotoxicity or cytokine production was observed to intestinal cells in vitro. Walczak et al., (2015) [92] observed that the translocation of positively charged polystyrene NMs was enhanced following simulated digestion. Büttner et al., (2022) [93] showed that the toxicity (barrier integrity and cytotoxicity) of undigested and digested copper oxide (CuO) NMs were comparable to intestinal cells in vitro. Lichtenstein et al., (2015) [94] also investigated what influence the presence of food components (carbohydrates, proteins and fatty acids) had on the toxicity of NMs to better mimic the in vivo ingestion scenario. It was observed that the uptake of Ag NMs by cells was increased when NMs were digested in the presence of food components, but there was no impact on cytotoxicity. Consequently, future studies could consider the physiological relevance of the dispersion protocol to a greater extent.

### Does a standard dispersion protocol exist that is applicable to all particles?

In this article we have assessed the evidence of whether various dispersion protocols affect the PC identity of particles (and in particular their agglomeration status), and in turn their toxicity. A vast range of dispersion protocols have been employed in the literature to prepare particle suspensions, and thus several standardised protocols have been proposed in attempt to bring harmonisation to the field. For example, the OECD (OECD, 2017 [74]) published a protocol tailored towards ecotoxicology hazard testing which contains aspects that may be applied to the dispersion of TiO_2_. The protocol assumes that the NM is supplied in dry powder form – which was generally the case in the literature assessed in Sect. 2. (Table 3) details the steps in the OECD dispersion protocol, including the benefit of each step and how these steps could be considered for dispersing TiO_2_.

**Table 3:** OECD dispersion protocol. Explanation as to why the OECD dispersion protocol includes specific steps, and how it aligns with the approach used in the published literature to help identify what steps May be important to consider when Preparing a dispersion protocol for TiO_2_

Dispersion step	Reasoning	Comment
A NM stock suspension is always prepared, from which samples are aspirated and diluted prior to delivery of the NM to the test specimen.	It is easier and more accurate to make one suspension per experiment from which other concentrations are diluted, than to make several different suspensions.	This is common practice in the publications identified.
The NM powder is pre-wetted in ultrapure water for 24 h to make a paste.	The OECD document suggests that this approach ensures proper interaction of NM surface with the water.	No details on the volume of water per mass of particle is provided. No details on the storage of the paste are provided. No comments are made on the impact of hygroscopic or water-soluble particles. It is not common practice to pre-wet NM powders for 24 h prior to use in the published literature
The recommended concentration of NMs within a stock dispersion shall not exceed a concentration > 20 X the NM within the analysed samples.	The greater the range of dilutions, the greater the chance for inaccuracies in the final particle concentration. Particle suspensions may not be homogenous or stable when produced and aliquoted to make subsequent dilutions.	Some publications are within the 20 dilution (e.g. 125 µg/ml – 6 µg/ml), but many go beyond this proposed limit.
The NM is dispersed in ultrapure water without the presence of any biological components.	Sonication of proteins can cause the proteins to be damaged.	Many studies prior to this recommendation suspended particles in media containing salts. Many such media also include biological components such as sugars, amino acids, antibiotics and serum.
The water volume (125 mL), vessel size (250 mL), and vessel type (e.g. glass beaker) are specified.	This is suitable for ecotoxicology testing where relatively large volumes of liquid are needed.	These volumes are excessive for in vitro human and in vivo rodent toxicity studies, where much smaller volumes are needed (typically < 2 mL). Sufficient amounts of particles may not be feasible for such dispersion protocols.
Probe sonication is applied to the NM suspension, using 40 W output power for 10 min.	Sonicators vary in power, and so by specifying the time and wattage, the amount of energy delivered can be standardised.	Many publications do not state the wattage or time or vary from this recommendation.
An ice bath is used during sonication.	Sonication can lead to heating of the sample which can impact on the particles (e.g. enhancing dissolution rate)	Many publications do not state whether cooling was used during or after sonication.
The NM dispersion is stored at 4 ^o^C (but do not freeze).	This allows the same suspension to be used across multiple experiments.	Careful characterisation would be needed to ensure the PC properties of the NM dispersion did not change with time. This would only be suitable for NMs with an extremely slow dissolution rate. No details are provided on the maximum amount of time that is suitable for storage. No justification or evidence is provided for this protocol step.
Following sonication (or storage), the NM suspension is diluted with biological media.	Components of the biological media may be damaged during storage	A vehicle control is also required that replicates the maximum amount of dispersion water included in the dispersion protocol.
The undiluted stock suspension can be stored for 14 d, but after more than 3 h a re-dispersion by probe sonication is required.	It is likely that particles will settle over time during storage.	Careful characterisation would be needed to ensure the NM dispersion PC did not change with time. This would only be suitable for NMs with an extremely slow dissolution rate. No details are provided on the maximum amount of time that is suitable for storage. No justification or evidence is provided for this protocol step. Retaining sterility for in vitro human cell cultures could be an issue.

The protocol described by the OECD shows some commonalities with that of the NANOGENTOX dispersion protocol (Jensen et al., 2011 [37]). Some of the noted similarities (that are specifically relevant to TiO_2_) include, preparation of the NM suspension in an aqueous media that does not contain biological components, the use of probe sonication (20 min at 40% power) with an ice bath, and the dilution of the NM suspension with biological media following sonication.

While such protocols outline a general dispersion protocol that can be applied to different NMs, we are limited in determining the reliability and effects of each of the dispersion steps on the impact on both NM agglomeration (as well as other PC properties) and toxicity, due to the variable uptake of these protocols by researchers. NM repositories, such as the JRC, can elucidate on the prediction of NM dispersion protocols for commercially obtained and ‘in-house’ developed materials alike. The JRC repository hosts NMs which originate from the same manufactured batch, where around 15 endpoints have been studied for each material, including many of the PC properties (e.g. chemical composition, size, shape, surface charge etc.). Identifying similarities between the PC properties of ‘in-house’ developed NM’s and standardised NMs from the JRC, may facilitate an entry point to tailor dispersion protocols. Correlations between these PC properties may permit prediction of agglomeration/aggregation of ‘in-house’ developed NMs thus minimising time to optimise new dispersion protocols. The JRC provides an already realised central database that research labs can access to minimise method development time.

## Discussion

For hazard studies on TiO_2_, many different sources of TiO_2_ were tested which varied with respect to their PC properties. Typically, stock suspensions of TiO_2_ used NM concentrations varying between 0.02 and 5 mg/mL [23, 47, 65, 67, 72]. TiO_2_ suspensions with a range of agglomerate sizes have been tested, however, the impact of such differences on toxicity was challenging to discern. For example, several studies discussed that smaller agglomerates of TiO_2_ exhibited an increase in genotoxicity and cytotoxicity in vitro [18, 23], while other studies suggest that larger agglomerates elicited a greater pro-inflammatory response in vitro and in vivo [18, 95].

Studies which used physiological dispersant media (e.g. cell culture media supplemented with various serums) generally showed that TiO_2_ agglomerate size decreased with the addition of FBS/FCS/BSA to cell culture media prior to sonication [31, 33, 57, 58]. Culture media supplemented with BSA however, showed both an increase [33, 58] and decrease [33] in NM size. Existing studies indicate that the addition of serum to the dispersant media can improve cell viability in cytotoxicity studies (both in the absence and presence of NMs), however, there is limited investigation on other toxicological markers, such as genotoxicity, proinflammatory response (acute and chronic) and oxidative stress. The physiological relevance of serum for dispersion should also be considered. Firstly, for lung studies (in vitro or in vivo) the relevance of serum can be questioned since serum proteins are not generally found on the lung surface (unless the lung is significantly damaged). In future, a simulant of lung lining fluid could be a suitable alternative for development and use. Furthermore, there is also a need to consider other opportunities to improve the physiological relevance of the dispersion protocol for other target sites (e.g. intestine), such as mimicking the transit of NMs through the GIT [96].

This review found that probe sonication was the most commonly applied dispersion strategy, with sonication times ranging between 30 s and 15 min, while in the case that bath sonication was used, the sonication time ranged between 15- and 30-min. Future studies should directly compare bath and probe sonication (at different sonication powers and durations). When performing probe sonication, the particle suspensions should be kept on ice. We would also recommend that this stock suspension should be prepared using water as the dispersant medium (as sonication can damage biological molecules (e.g. proteins)), however a comparison between water and biological media should be performed to ascertain the extent to which TiO_2_ PC properties and toxicity are influenced by the media choice. When performing this work variables such as particle concentration, sonication power and duration can be informed from standard protocols produced by NANOGENOTOX and OECD [37, 74].

Whilst it has been speculated that sonication can enhance ROS production in NM suspensions there is a lack of experimental evidence to support this hypothesis. It is likely that the lifetime of ROS is relatively short (seconds or less), which means that these ROS are unlikely to drive the toxic responses that are observed. However, this has not been investigated. It would therefore be useful to assess ROS production immediately after sonication of the stock suspension in water, at regular time intervals in this water suspension, following dilution in the biological media in the culture media in the conditions used for the experimental study. One other theoretical source of ROS could be the release of surface moieties on modified NMs caused by sonication that may themselves be ROS or interact with biological components within the dispersant medium that in turn produce ROS. Studies are therefore needed to explore the role of sonication mediated ROS production to TiO_2_ toxicity.

Existing studies often fail to assess the impact of the dispersion protocol on *BOTH* the PC identity and toxicity of NMs. Thus, we suggest this knowledge gap is addressed in parallel in the future. It is recommended that the following PC properties of TiO_2_ should be assessed to identify the impact the dispersion protocol has had on the PC identity of the material; agglomeration, hydrodynamic diameter, particle size distribution, shape, surface charge, and if possible, surface properties and solubility. Assessment of toxicity of the TiO_2_ suspensions prepared using different dispersion protocols should be performed. This assessment should start with in vitro hazard studies, focussing on cytotoxicity, genotoxicity, oxidative stress and pro-inflammatory responses in cell types relevant to the expected route of exposure to TiO_2_. It is recommended that the stock suspensions should not be stored prior to testing, to ensure the stability of the suspension is not affected and as transformations to the PC identity of particles can occur during storage.

While key variables of the dispersion that affected agglomeration (i.e. primary particle size, particle concentration, dispersant media, method of sonication and sonication time) have been identified, it is recommended that a stepwise approach to developing a dispersion protocol is followed [28], where testing of the effect on changes of each step to the pristine NM is conducted. Such an approach would permit gathering of quantitative data, at the point of introducing a new variable into the dispersion protocol, facilitating the development of a more relevant dispersion protocols.

As such, it is recommended that dispersion protocols are tailored towards the test material, the biological test model, exposure system and the aim of the study [28]. While this has not yet been widely discussed, we predict that in the future standard dispersion protocols will not be generated, and instead more guidance will be provided on how to develop a dispersion protocol that is relevant to the particle and test model under investigation. It is also likely that researchers will be asked to justify their approach to particle dispersion to a greater extent, including acknowledgement of the strengths and limitations, the physiological relevance and how the protocol could influence the PC identity and toxicity of the test material. (Table 4) provides a summary of the recommendations suggested in this review.

**Table 4:** Summary of recommendations

Dispersion protocols should be tailored towards the test material, the biological test model, and the aim of the study [28].	Overall recommendations
Studies should assess the impact of the dispersion protocol on *BOTH* the PC identity and toxicity of NMs simultaneously.	
Based on current information, we would recommend that the stock suspension should be prepared using water as the dispersant medium (as sonication can damage biological molecules e.g. proteins).	Recommendations for specific dispersion steps
However, in future a comparison between water and biological media should be performed to ascertain the extent to which TiO_2_ PC properties and toxicity are influenced by the media choice.	
Future studies should directly compare bath and probe sonication (at different sonication powers and durations).	
When performing probe sonication, the particle suspensions should be kept on ice.	
The stock suspensions should not be stored prior to testing, due to the risk of transformation over time.	
For dispersions to be used for pulmonary toxicity studies, the impact of substituting a simulant of lung lining fluid for the usual serum ingredients could be assessed for relevance and suitability to retain cell viability and function.	
For dispersions to be used for gastrointestinal toxicity studies, a protocol that mimics the transit of NMs through the GIT (96) prior to addition to cell cultures could be employed (100).	
Studies are required to assess ROS production immediately after sonication of the stock suspension in water, at regular time intervals in this water suspension, following dilution in the biological media in the culture media in the conditions used for the experimental study.	Recommendations for ROS
The following PC properties of TiO_2_ should be assessed to identify the impact of the dispersion protocol - agglomeration, hydrodynamic diameter, particle size distribution, shape, surface charge, and if possible, surface properties and solubility.	Recommendations for PC characterisation
Assessment of toxicity of the TiO_2_ suspensions prepared using different dispersion protocols should be performed. This assessment should start with in vitro hazard studies, focusing on cytotoxicity, genotoxicity, oxidative stress and pro-inflammatory responses in cell types relevant to the expected route of exposure to TiO_2_.	Recommendations for toxicity assessment
Rather than providing a standard dispersion protocol for all NMs, we recommend generating guidance on how to develop such a protocol that is relevant to the particle and test model under investigation. The dispersion protocol should include: • Justification for the approach used. • Acknowledgement of the strengths and limitations of the approach used. • Clarification of the physiological relevance to the route of exposure. • PC characterisation data of the dispersed NM. • How the protocol could influence the PC identity of the NM.	Recommendations for future protocol content

In this review, we identified that the majority of studies aimed to mimic lung exposure when assessing the hazard profile of TiO_2_. Data gathered from several sources on airborne TiO_2_ released in workplace activities during the manufacture of TiO_2_ [97–99], revealed that depending on the work activity, released NM sizes ranged from < 100 nm to > 100 nm. It was reported that TiO_2_ typically agglomerated/aggregated in the workplace air. Kaminski et al., (2015) [99], gathered data from a production plant, finding that during the production/ handling / bagging process, 70–87% of the particle number concentration was attributable to particles ≤ 100 nm. Developing dispersion protocols which accurately reflect different sized particles/agglomerates is likely necessary to ascertain reliable toxicology data. This affirms the need to ensure that dispersion protocols are properly reported, and that NM physico-chemical properties are well-documented prior to commencing toxicology assays.

## Conclusion

An understanding of how the dispersion protocol effects both PC identity and toxicity is required to better understand the adverse effects of a particle to human health and the environment. Such an approach allows an understanding of how toxicity is impacted by both intrinsic (e.g. primary particle size, composition) and extrinsic properties (e.g. agglomeration, dissolution) due to external variables (e.g. dispersion protocol). This was confirmed by this critical review of the literature which found that NM agglomeration is impacted by the dispersion protocol, which can influence toxicity. There is no consensus regarding whether smaller agglomerates are more toxic than larger agglomerates and so this knowledge gap needs to be addressed. Whilst the details of the experimental design required for a robust reliability assessment lacked in many of the published studies, leading to a Klimisch Score of 3 and a low Nano Score, it was obvious publications rarely focussed on relating the effects of the dispersion protocol on both NM PC identity and toxicity. Through our analysis, it is evident that it is unlikely to be possible to develop a standard dispersion protocol that is applicable to all forms of TiO_2_ and all biological experimental models. However, we have identified aspects of the dispersion protocol that appear to be particularly influential in impacting on the PC identity and toxicity of particles, such as the media that particles are suspended in (including the use of biological dispersants), sonication type, power and duration, storage time, temperature and exposure of TiO_2_ suspension to light during preparation. Further assessment into variables of particle dispersion such as a comparison between sonication methods, or dispersant mediums with and without biological components, will aid development of tailored protocols for TiO_2_ dispersion. Since different forms (e.g. composition or coating) of TiO_2_ are available, it is possible that more than one dispersion protocol may be required, or perhaps guidance on how to generate or identify the most relevant protocol. Therefore, the testing of different forms of TiO_2_ and dispersion protocols relevant to a range of exposure routes is required to ensure toxicity studies are testing a form of the material that is representative of the human’s exposure.

## Electronic supplementary material

Below is the link to the electronic supplementary material.

Supplementary Material 1: Additional file 1: Table S1. Impact of NM agglomeration on NM toxicity. An assessment of the Klimisch- and Nano- Score is provided for each identified study.

Supplementary Material 2: Additional file 2: Table S2. Summary on the reporting of the influence of dispersant media on NM PC and toxicity. Data summarising test material, dispersion protocol, dispersant media, including Nano Score and Klimisch Score of research articles which report the influence of dispersant media on NM PC and toxicity.

Supplementary Material 3: Additional file 3: Table S3. Summary on the reporting of the influence of sonication on NM PC identity and toxicity. Data summarising test material, dispersion protocol including Nano Score and Klimisch Score of research articles which report the influence of sonication on NM PC and toxicity.

Supplementary Material 4: Additional file 4: Table S4. Summary on the reporting of the influence of the storage conditions of NM stock suspensions on NM PC identity and toxicity. Data summarising test material, dispersion methodology, storage temperature including Nano Score and Klimisch Score of research articles which report the influence of the storage conditions of NM stock suspensions on NM PC and toxicity.

## Data Availability

Data is provided within the manuscript or supplementary information files.

## Abbreviations

ALI: Air Liquid Interface
BALF: Bronchoalveolar Lavage Fluid
BSA: Bovine Serum Albumin
CNTs: Carbon Nanotubes
DLS: Dynamic Light Scattering
ENPRA: Risk Assessment of Engineered Nanoparticles
EPR: Electron Paramagnetic Resonance
FBS: Fetal Bovine Serum
FCS: Fetal Calf Serum
GIT: Gastrointestinal Tract
H&E: Haematoxylin and Eosin
HBSS: Hank’s Balanced Salt Solution
HAS: Human Serum Albumin
NM: Nanomaterial
NOM: Natural Organic Matter
OECD: Organisation for Economic Co-operation and Development
PBS: Phosphate Buffered Saline
PC: Physico-chemical
ROS: Reactive Oxygen Species
SEM: Scanning Electron Microscope
ToxRTool: Toxicological Data Reliability Assessment Tool
TEM: Transmission Electron Microscopy
US: Ultrasound
XRD: X-Ray Diffraction Analysis
