# Supplementary material for: Investigating the impact of the dispersion protocol on the physico-chemical identity and toxicity of nanomaterials: a review of the literature with focus on TiO2 particles
Source: Part Fibre Toxicol. 2025 May 13;22:11. doi: 10.1186/s12989-025-00627-8 (PMC12070512; doi:10.1186/s12989-025-00627-8)
Supplement: Supplementary file 1 — Supplementary Material 1: Additional file 1: Table S1. Impact of NM agglomeration on NM toxicity. An assessment of the Klimisch- and Nano- Score is provided for each identified study. [file 12989_2025_627_MOESM1_ESM.docx]

**Additional file 1**

**Table S1: Impact of NM agglomeration on NM toxicity.** An assessment of the Klimisch- and Nano- Score is provided for each identified study.

| **Study Ref.** | **Test material**  (Supplier Information) | **Individual NM size** | **Nano Score (0-10)** | **Agglomerate/ Aggregate size** | ***In vitro/in vivo* model used** | **Dosage information** | **Toxicity effects: significant results** |
| --- | --- | --- | --- | --- | --- | --- | --- |
| **Klimisch Score = 1** | | | | | | | |
| Gosens et al., 2010 (21) | Au  2 sizes (50 nm, 250 nm)   Supplier:  BBI International | 61 ± 3 and 197 ± 12 nm  Measured by researchers: laser illuminated microscopic technique and TEM. | 8 | 199 ± 88 and 768 ± 766 nm  Measured by researchers: laser illuminated microscopic technique and TEM. | Rat, male | Single exposure  Intratracheal instillation, 1.6mg/kg (BW of rat) of Au particles in suspension  Exposure duration: 3 and 24 | Both single and agglomerated particles were taken up by macrophages. Single and agglomerate particles elicited small increases in inflammatory cell accumulation, pro-inflammatory cytokine production and acute phase protein expression.  **No significant differences in pro-inflammatory cytokine production between single and agglomerated particles.** |
| **Klimisch Score = 2** | | | | | | | |
| no studies identified | | | | | | | |
| **Klimisch Score = 3** | | | | | | | |
| Noël et al., 2013 (95) | TiO_2_  3 sizes (5, 10-30, 50 nm)  Supplier:  Nanostructured and Amorphous Materials Inc, and MKnano | Median sizes: 10.6, 18.2 and 34.8 nm  Measured by researchers:  TEM and XRD | 5 | Small aggregates (SA) < 100 nm  Large aggregates (LA) > 100 nm  Measured by researchers:  TEM | Rat, male | Single exposure  Inhalation, aerosol concentration 20 mg/m^3^, exposure duration = 6 h | SA exhibited high oxidative stress damage (highest for 10-30 and 50 nm particle sizes), and increased LDH release.  LA induced the greatest acute inflammatory response via increased number of neutrophils (greatest increase with 10-30 and 50 nm particle sizes).  No increase in other inflammation markers (IL-1α, IL-6, and TNF-α production) was observed.  **SA NMs found to be more genotoxic, while LA NMs elicited a greater inflammatory response**. |
| Peng et al., 2014 (22) | CeO_2_  1 size (3 - 5 nm)  Supplier: synthesised in-house | No individual characterisation conducted | 4 | For CeO_2_ -p:  313 ± 30 nm  For CeO_2_ -h:  1731 ± 165 nm  Measured by researchers:  TEM | Mouse, male | Single exposure  Inhalation, 50 µL of particle suspension, 0.8 mg/mL  Animals were sacrificed at 1-, 3-, 7- and 28- days post-instillation. | Both sizes of NMs caused similar levels of oxidative stress across 28 days.  CeO_2_ -p (smaller aggregates): high acute (1 – 3 days) inflammation response (pronounced neutrophil production) compared to control and the larger aggregates.  CeO_2_ -h (larger aggregates): high inflammatory response, greater than smaller aggregates at day 28 (I-6 marker used).  **Smaller aggregates exhibited a higher acute inflammatory response, while larger aggregates elicited a greater immune response at later stages.** |
| Sharma et al., 2014 (19) | Fe_2_O_3_  1 size (12.8 nm)  Supplier: synthesised in-house | 12.8 nm  Measured by researchers:  TEM | 5 | Carboxyl modified aggregates:  276 ± 6 nm  689 ± 43 nm  Amine modified aggregates:  360 ± 15 nm 1463 ± 93 nm  Measured by researchers:  TEM and SEM | Cell Line: C10 alveolar epithelial line  (mouse) | Cell viability: 5, 10, 50, 100 and 200 µg/mL, exposure duration = 24 h  Stress-related gene induction/ Exposure- and time-dependent delivery of NM agglomerates: 10, 25, 50 and 100 mg/ml, exposure duration = 4 h | Small agglomerates of  carboxylated Fe_2_O_3_ induced greater cytotoxicity and redox regulated gene expression when compared with large agglomerates (equivalent total cellular Fe_2_O_3_ mass dose).  Agglomerates of amine modified Fe_2_O_3_ failed to induce cytotoxicity or redox-regulated gene expression despite delivery of similar cellular doses.  **Smaller aggregates were found to be more cytotoxic and genotoxic compared to larger aggregates.** |
| Hu et al., 2019 (18) | TiO_2_  2 sizes (21 and 35 nm)  Supplier: Sigma Aldrich | No individual characterisation conducted | 7 | For 21 nm:  352 nm  For 35 nm:  483 nm  Measured by researchers:  TEM | Cell Line: RAW264.7 (mouse) | Exposure duration=48h  Cell viability/LDH release: 0.1-10^-9^ mg/mL  ROS generation/ mitochondria dysfunction, cell autophagy, and immune activation response of macrophages:  0.1-10^-5^ mg/mL | Smaller aggregates (21 nm) exhibited higher cytotoxicity, which correlated with their ability to cause damage to mitochondria compared to larger aggregates.  Larger aggregates (35 nm) exhibited higher level of cell autophagy, stronger pro-inflammatory immune response and lower cytotoxicity compared to smaller aggregates.  **Smaller aggregates were found to be more cytotoxic, while larger aggregates elicited a greater immune response.** |
| Murugadoss et al., 2020 (16) | TiO_2_  2 sizes (17 and 117 nm)  Supplier:  European Commission’s Joint Research Centre | Median in solution:  18 and 122 nm.   Measured by researchers: TEM | 4 | For 17 nm; smaller aggregates (SA) = 600 nm, larger aggregates (LA) = 900 nm  For 117 nm; SA = 280 nm, LA = 580 nm  Measured by researchers:  TEM and DLS | Mouse, female | Single exposure  Inhalation, 50 µL of particle suspension, dose 0-50 µg  Oral gavage,  200 µL of particle suspension  Animals sacrificed after 3 d. | Inhalation  For 117 nm particles: LA induced increased BALF lymphocytes compared to SA. LA exhibited the greatest persistence in lungs.  For 17 nm particles: no such differences between agglomerate sizes observed.  Oral Gavage  For 117 nm particles: LA induced higher blood DNA damage than SA.  **Greater difference in toxicity observed between SA and LA for aggregates of 117 nm particles compared to 17nm particles (*in vivo*). LA generally found to be more toxic.** |
| Murugadoss et al., 2020 (16) | TiO_2_  2 sizes (17 and 117 nm)  Supplier:  European Commission’s Joint Research Centre | Median in solution:  18 and 122 nm.   Measured by researchers: TEM. | 4 | For 17 nm; smaller aggregates (SA) = 600 nm, larger aggregates (LA) = 900 nm  For 117 nm; SA = 280 nm, LA = 580 nm  Measured by researchers:  TEM and DLS | Cell Lines: HBE, THP-1, Caco2  (human) | Exposure duration = 24 h  For cell cytotoxicity, total glutathione, epithelial barrier integrity, inflammatory mediators:  dose = 4 to 256 μg/mL  For DNA damage: dose = 5 - 10 μg/mL | For 17 nm particles: LA induced stronger responses than SA for glutathione depletion, IL-8 and IL-1β increase, and DNA damage in THP-1 cells.  For 117 nm particles: No observed differences between LA and SA for each assay, regardless of cell line.  **Greater difference in toxicity observed between SA and LA for aggregates of 17 nm particles compared to 117 nm particles (*in vitro*). LA generally found to be more toxic.** |
