# Supplementary material for: Investigating the impact of the dispersion protocol on the physico-chemical identity and toxicity of nanomaterials: a review of the literature with focus on TiO2 particles
Source: Part Fibre Toxicol. 2025 May 13;22:11. doi: 10.1186/s12989-025-00627-8 (PMC12070512; doi:10.1186/s12989-025-00627-8)
Supplement: Supplementary file 2 — Supplementary Material 2: Additional file 2: Table S2. Summary on the reporting of the influence of dispersant media on NM PC and toxicity. Data summarising test material, dispersion protocol, dispersant media, including Nano Score and Klimisch Score of research articles which report the influence of dispersant media on NM PC and toxicity. [file 12989_2025_627_MOESM2_ESM.docx]

**Additional file 2**

**Table S2:** **Summary on the reporting of the influence of dispersant media on NM PC and toxicity.**

| **Study Ref.** | **Test Material** | **Dispersion Protocol** | **Dispersant Medium** | **Model System incl. Exposure Details** | **Nano Score (0-10)** | **Reported Findings** |
| --- | --- | --- | --- | --- | --- | --- |
| **Toxicity of NM not assessed – no Klimisch Score** | | | | | | |
| Bihari et al., 2008 (31) | TiO_2_ primary NM size = 10 x 40 nm  Size characterisation provided by suppliers | Probe sonication (energy = 4.2 × 10^5^ kJ/m^3^) NM concentration = 0.02 mg/mL.  Volume = 870 µL. | RPMI 1640 RPMI 1640 + HSA (30 µL) RPMI 1640 + Tween 80 (30 µL) RPMI 1640 + mouse serum (30 µL) | N/A: impact on PC identity assessed. | 5 | TiO_2_ particles dispersed in PBS have a higher average diameter and zeta potential increased from ~ -40 to -19 mV compared to dispersion in cell culture medium.  HSA or Tween 80 added to the TiO_2_ dispersion (without previous sonication) did not reduce the particle diameter in PBS. However, the  TiO_2_ particle diameter was reduced in PBS when HSA or Tween 80 was added to the dispersion (after sonication). Similar results were observed when HSA was replaced with mouse serums. |
| Schulze et al., 2008 (46) | ZrO_2_ primary NM size = 14 nm  TiO_2_ primary NM size = 21 nm CeO_2_ primary NM size = 30 nm  Size characterisation provided by suppliers | Stock suspensions were stirred (24h) and then 900 rpm for 1h.  NM concentration = 1-10g/L.  Volume not reported. | H_2_O Phosphate buffer Isotonic phosphate buffer Krebs-ringer buffer DMEM DMEM+FCS | N/A: impact on PC identity assessed. | 8 | NMs agglomerate sizes showed an increase in phosphate and Krebs-ringer buffers compared to water. Addition of CaCl_2_ to all buffers resulted in an increase in agglomerate size. The use of Krebs-ringer buffer resulted in larger sized agglomerates compared to phosphate buffers considered to be due to a high Ca^2+^ concentration.  1% BSA added to Krebs buffer resulted in complete de-agglomeration.  Zeta-potentials of CeO_2_ and ZrO_2_ in millipore water shifted from positive to negative values in DMEM containing 10% FCS.  Adding FCS to DMEM resulted in de-agglomeration of NMs. |
| Vippola et al., 2009 (33) | TiO_2_ (rutile)  primary NM size = 10 x 40 nm TiO_2_ (anatase) primary NM size = < 25nm  Carbon nanotubes (single walled) primary NM size = outer diam. < 2 nm, length 1 - 5 μm  Carbon nanotubes (single walled) primary NM size = outer diam. < 2 nm, length 1 - 5 μm  Carbon nanotubes (multiwalled) primary NM size = outer diam. 10 - 30 nm, length 1 - 2 μm  Size characterisation provided by suppliers. | Bath sonication for 30 min.  NM concentration = 1mg/mL.  Volume not reported. | BEGM media BEGM + BSA BEGM + BSA+ DPPC (phospholipid)  BEGM + DPPC  RPMI + FCS RPMI + FCS + BSA RPMI + FCS+BSA DPPC RPMI +FCS+DPPC | N/A: impact on PC identity assessed. | 5 | BEGM media:  Addition of BSA to BEGM resulted in a decrease in NM size for all NMs. BEGM +BSA+DPPC, resulted in an increase in NM size compared to BEGM+BSA for all NMs.  RPMI media: RPMI+FCS+BSA resulted in increase in NM size compared to RPMI+FCS for all NMs. RPMI+FCS+DPPC resulted in an increase in NM size compared to RPMI +FCS for all NMs. |
| Orts-Gil et al., 2011 (58) | SiO_2_ primary NM size = ~ 30 nm  NM size characterised by researchers | Bath sonication used; time not specified.  NM concentration ranges from 0.1-10 wt%.  Volume not reported. | NaCl solutions  DMEM DMEM + 10 % FCS Aqueous solution + BSA (1 – 3 %) | N/A: impact on PC identity assessed. | 5 | When concentrations of NaCl above 0.5 M were used, particles showed an increase in the average size.  Zeta potential decreased when increasing the BSA content, while particle size increased.  Addition of FCS to DMEM decreases the particle size compared to DMEM without added serum. |
| Izak-Nau et al., 2013 (45) | SiO_2_/SiO_2__NH_2_/ SiO_2__SH/ SiO_2__PVP primary NM size = 50 nm  Size characterisation provided by suppliers | Dispersion method not reported.  NM concentration = 2% (wt/wt); 1.5x10^14^ NM /mL  Volume not reported | H_2_O PBS  DMEM DMEM + 10 % FBS | N/A: impact on PC identity assessed | 7 | All of the studied SiO_2_ NMs tended to agglomerate after relatively short time periods in all buffers and biological media.  Agglomeration was diminished in a medium containing serum.  The protein corona formation depended on time and functionalization of the NM and varied significantly in different types of serum.  NMs which attracted the highest amount of FBS proteins were the plain SiO_2_ and the SiO_2__NH_2_. |
| Freyre-Fonseca et al., 2016 (57) | TiO_2_ primary NM size = < 25 nm 3 nanoforms: spheres, E171, belts  Size characterisation provided by suppliers | Bath sonication for 30 min.  NM concentration = 1mg/mL  Volume = 1mL.  2.4L of the stock suspension was the aliquoted into cell culture media to reach a NM concentration of 3.43×10^−3^ mg/𝜇L. | H_2_O FBS  F12K  F12K + FBS | N/A: impact on PC identity assessed. | 6 | All agglomerates exhibited larger diameters compared with the initial sizes over 24 h.  Smallest sizes of agglomerates were found for NM forms spheres and belts in FBS media.  The zeta potential for dispersions of the NM form E171 was higher than that for NM belts and spheres.  Zeta potential was higher for all nanoforms dispersed in F12K without serum compared to with serum.  Circularity of the agglomerates of spheres in FBS and H_2_O differed, but there were no significant differences in these values for the agglomerates formed by E171 NM and belts in the same media or for the agglomerates formed for the same NM in F12K and in F12K + FBS. |
| **Klimisch Score = 1** | | | | | | |
| Henry et al., 2007 (54) | C60  Primary NM size = not assessed  Size characterisation not provided (agglomerate size provided by researchers – 50 - 300 nm) | NMs were stirred for 7 days then sonicated ( no details supplied).  NM concentration = 40 mg/mL.  Volume = 500 mL. | H_2_O  THF | Zebrafish larvae (age 75 h)  Duration of exposure = 72 h  Concn 1 – 25 % vol/vol | 2 | Survival of larval zebrafish was reduced in THF–C60 and THF–water but not in C60–water. The greatest differences in gene expression were observed in fish exposed to THF–C60 and most (182) of these genes were similarly expressed in fish exposed to THF–water.  Significant up-regulation (3- to 7-fold) of genes involved in controlling oxidative damage was observed after exposure to THF–C60 and THF–water. THF oxidation products γ-butyrolactone and tetrahydro-2-furanol present toxic effects attributed to THF degradation products rather than C60. |
| **Klimisch Score = 2** | | | | | | |
| no studies identified | | | | | | |
| **Klimisch Score = 3** | | | | | | |
| Murdock et al., 2008 (30) | Al_2_O_3_ primary NM size = 30 and 40 nm  Al primary NM size = 80 and 120 nm Cu  primary NM size = 40, 60 80 nm SiO_2_ primary NM size = 35, 51, 110, 420 nm TiO_2_ primary NM size = 39 nm (various anatase: rutile ratios)  Size characterisation provided by suppliers. | NMs were probe sonicated for 30 s (35 W)  NM concentration = 25–50 μg/mL.  Volume not reported. | H_2_O RPMI RPMI + 10% FBS RPM + 10% HI F-12K F-12K + 10% FBS F-12K+ 10% HI F-12K+ 20% FBS DMEM/F-12 DMEM/F-12 + 10% FBS | HEL-30 (mouse)   Duration of exposure = 24 h  Concn  25 or 50 μg/mL | 5 | Al_2_O_3_ NMs agglomerated highly in DMEM-F12K compared to water, with a decrease in agglomeration when serum was added to DMEM-F12K.  All the Al-based NMs tended to form agglomerates of similar size when dispersed in either water or cell culture media.  Cu NMs exhibited increased agglomeration in media and decreased agglomeration in media with serum.  SiO_2_ NMs exhibited slightly higher agglomeration in media compared to water.  TiO_2_ NMs exhibited similarly high agglomeration in water/media/media + serum.  Cell viability decreased with exposure to NMs, however, the addition of serum to media either did not change or increase cell viability compared to cells exposed to the serum-free suspensions. |
| Drescher et al., 2011 (48) | SiO_2_ primary NM size = 38 nm  NM size characterised by researchers | Method of dispersion not stated.  NM concentration not reported.  Volume not reported. | DMEM DMEM + 10% FCS | 3T3 (mouse)  Duration of exposure =  24 h  Concn.  5 to 100 μg/mL | 3 | In DMEM supplemented with FCS, increase in particle size is observed due to agglomeration of silica particles.  Cell viability decreased as function of silica concentration.  DMEM + 10% FCS improves cell viability compared to media without serum proteins. |
| Magdolenova et al., 2012 (47) | TiO_2_ primary NM size = 21 nm  Size characterisation provided by suppliers. | Protocol 1:  Probe sonication for 15 min.  NM concentration = 5 mg/mL Volume = 1 mL Stock suspension then diluted with cell culture medium to achieve a NM concentration of 75 µm/cm^2^  Protocol 2: Probe sonication for 3 min.  NM concentration = 2 mg/mL Volume = 10 mL Stock suspension was then vortexed for 10 s, sonicated for 1 min then diluted with cell culture medium to achieve a NM concentration of 75 µm/cm^2^ | Protocol 1:  PBS+ 20% FBS, then diluted with culture media (RPMI or DMEM).  Protocol 2: HEPES buffer, then diluted with culture media ((RPMI or DMEM). | TK6  (human)  Cos-1  (monkey) EUE (human)  Duration of exposure = 2, 24, 72 h  Concn.  0 - 75 µg/cm^2^ | 9 | Protocol 1 using FBS resulted in relatively stable dispersions of TiO_2_ NMs, while the use of Protocol 2 resulted in the rapid formation of TiO_2_ NM agglomerates.  No significant DNA damage was observed in TK6/Cos-1/EUE cells using Protocol 1 (media with serum) after 2 and 24 h.  Using protocol 2 (no serum), DNA strand breaks were not observed in TK6 cells following NM exposure after 2 or 24 h, however, significant strand breaks were observed for Cos-1/EUE cells at the highest concentration of TiO_2_.  TiO_2_ did not affect trypan blue exclusion or proliferation assays for TK6 cells, when the NM suspension was prepared with either protocol.  TiO_2_ dispersed using Protocol 2 was cytotoxic to Cos-1 cells at concentrations between 15-75 µm/cm^2^. For EUE cells, TiO_2_ did not inhibit proliferation activity using the Protocol 1 dispersion method, however using Protocol 2, a slight decrease in proliferation was observed at the two highest concentrations of NM. |
| Gutierrez et al., 2015 (84) | NiO  primary NM size = < 20 nm  Size characterisation provided by suppliers. | Bath sonication for 30 min.  NM concentration = 0.01 mg/mL.  Volume = 100 mL | F-12K + FBS BSSM media F-12K+ Pluronic (F68) MEM+ FBS MEM+ Pluronic (F68) | A549 and 16hbe14o  (human)  Duration of exposure = 4 and 24 h  Concn.  0, 0.01, 0.1,  0.5, 10, 100 µg/mL | 4 | NiO NM agglomerate size increases when pluronic is added to media + serum.  In BSSM, agglomerate size varies with NM concentration. NM size is higher than F12K + pluronic for concentrations below 0.5 ug/mL, while the opposite is seen for higher NM concentrations.  ELISA assays showed all three dispersion media formulations produced significantly lower concentrations of IL-8 compared to the control, but no difference between dispersant media was found. High variability in HO-1 (oxidative stress marker) was found in data collected across the different dispersant media. HO-1 expression increased at 24 h compared to 4 h. |
