# Supplementary material for: Investigating the impact of the dispersion protocol on the physico-chemical identity and toxicity of nanomaterials: a review of the literature with focus on TiO2 particles
Source: Part Fibre Toxicol. 2025 May 13;22:11. doi: 10.1186/s12989-025-00627-8 (PMC12070512; doi:10.1186/s12989-025-00627-8)
Supplement: Supplementary file 3 — Supplementary Material 3: Additional file 3: Table S3. Summary on the reporting of the influence of sonication on NM PC identity and toxicity. Data summarising test material, dispersion protocol including Nano Score and Klimisch Score of research articles which report the influence of sonication on NM PC and toxicity. [file 12989_2025_627_MOESM3_ESM.docx]

**Additional file 3**

**Table S3: Summary on the reporting of the influence of sonication on NM PC identity and toxicity.**

| **Study Ref.** | **Test Material** | **Dispersion Protocol:   Nanomaterial stock suspension** | **Dispersion Protocol:   Method of Dispersion** | **Model System incl. Exposure Details** | **Nano Score (0-10)** | **Reported Findings** |
| --- | --- | --- | --- | --- | --- | --- |
| **Toxicity of NM not assessed – no Klimisch Score** | | | | | | |
| Nguyen et al., 2011 (68) | Al_2_O_3_ primary NM size = 13 nm  Size characterisation provided by suppliers. | Particle concentration 1 mg/mL.  NMs dispersed in 10 mL of deionized water. | Probe sonication.  Pulsed sonication with the pulse ratio on/off 0.1/2.0 (s/s) was performed at room temperature without any cooling systems. For the other pulse ratios on/off 0.1/0.1 (s/s), 1.0/1.0 (s/s) and continuous mode, the processing sample was cooled using an ice-water bath.  Sonication duration was 180s. | N/A: impact on PC identity assessed. | 3 | Agglomerate size reduced as the sonication amplitude increased from 10 – 30 %. Higher vibrational amplitudes showed no improvement in the breakage process, while prolonged sonication resulted in the reagglomeration of NMs.  Under optimal conditions, both continuous and  pulsed irradiation showed almost the same efficiency of deagglomeration over a specified time. |
| Tajik et al., 2012 (69) | Al_2_O_3_ primary NM size = 27 – 43 nm  Size characterisation provided by suppliers. | Particle concentration not specified.  NM dispersed in deionized water.  Volume not reported. | Probe sonication for 30 min.  Continuous sonication: 100% amplitude.  Discontinuous sonication: 50% amplitude. | N/A: impact on PC identity assessed. | 2 | Continuous sonication resulted in a higher degree of deagglomeration compared to discontinuous sonication. |
| Nickel et al., 2014 (70) | TiO_2_ primary NM size = 21 nm  Size characterisation provided by suppliers | Particle concentration = 100 mg/L  NMs dispersed in HPLC grade water supplemented with 1 % sodium hexa-methaphosphate | Either bath or probe sonication were used. Methodologies varied greatly. | N/A: impact on PC identity assessed | 4 | The study assessed comparability between using different DLS instruments (across different laboratories) to measure the variability in NM particle size. No significant differences between the employed DLS instrument types were found.  TiO_2_ agglomerates exhibited similar sizes (211 ± 11 nm) when suspensions were prepared at each laboratory followed an established dispersion protocol. |
| Pradhan et al., 2016 (24) | Cu: primary NM size = 100 ± 34 nm.  Al: primary NM size = 70 ± 26 nm.  Mn: primary NM size = 20 ± 7 nm.  ZnO: primary NM size = 120 ± 90 nm.  All sizes characterised by researchers | NMs are prepared in scintillation vials. Particle concentration = 1 or 2.56 g/L. NMs dispersed in ultrapure water +/- 0.05 % BSA.  Volume not reported. | Probe sonication: 3- or 15-min. Cooling system used. Sonicator had 400 W output power.  US bath: 15 min sonication.  Following sonication, either bath or probe, suspensions were diluted to 0.1 g/L in 1 mM NaClO_4_. | N/A: impact on PC identity assessed. | 4 | Probe sonication resulted in a decreased mean agglomerate size compared to bath sonication.  Probe sonication for 3 min resulted in larger homogeneously sized agglomerates, while prolonged probe sonication results in smaller agglomerate sizes, slightly increased metal dissolution and no significant effect on the zeta potential.  Addition of BSA increased the release of metal ions in sonicated samples. |
| Cohen et al., 2018 (66) | Ag15%/SiO_2_: primary NM size = 6.4 nm (Ag)  6.1 ± 0.1 nm (SiO_2_)  Ag:  primary NM size = 18.3 ± 0.3 nm  CeO_2_:  primary NM size = 5.3 ± 0.2 nm  Fe_2_O_3_:  primary NM size = 10.2 ± 0.5 nm  All sizes characterised by researchers | NM are prepared in conical, polyethylene tubes. Particle concentration = 500 µg/ml. NMs dispersed in deionized water, 50 mL. | Cup and horn sonication. Discrete sonication: sonication energy delivered in short intervals, interspersed with periods of vortexing. Continuous sonication: sonicating the suspension without stopping until delivered sonication energy reached (For Ag15%/SiO_2_ = 480J/mL, Ag = 350J/mL, CeO_2_ = 630J/mL, Fe_2_O_3_ = 320J/mL).  Sonicator power  output = 2.59 W.  Temperature not reported. | N/A: impact on PC identity assessed. | 4 | Fast settling NMs were identified as Ag15%/SiO_2,_ Ag and CeO_2_, while a slow settling NM was shown to be Fe_2_O_3_ NMs.  The discrete sonication protocol was found to achieve a significant reduction in the agglomerate diameter and polydispersity for fast settling NMs. However, no statistically significant difference in agglomerate size or polydispersity was found for the slow settling NMs, when comparing the continuous and discrete sonication protocols.  Fe_2_O_3_ required the lowest delivered sonication energy to achieve de-agglomeration. |
| **Klimisch Score = 1** | | | | | | |
| no studies identified | | | | | | |
| **Klimisch Score = 2** | | | | | | |
| no studies identified | | | | | | |
| **Klimisch Score = 3** | | | | | | |
| Cronholm et al., 2011 (36) | Cu  primary NM size = 100 nm.  Size characterisation provided by suppliers. | NM concentration = 1 mg/mL. Serum-containing medium.  2mL suspension. | 20 s vortex, followed by 2 x 20 s in US bath, with 20s pause in between.  Temperature not reported. | A549  (human)  Duration of exposure = 4 h.  Concn.  <80 µg/mL | 4 | Increase in Cu ion release observed when NMs suspension were sonicated compared to non-sonicated samples.  Sonication of Cu NMs resulted in an observed decrease in cell viability compared to non-sonicated samples.  Sonication had no effect on observed DNA strand breaks or oxidative lesions. |
| Hamzeh & Sunahara, 2013 (23) | TiO_2_ primary NM size = 5.9 - 169 nm  NM size characterised by researchers | NM concentration = 200 mg/L.  Serum-containing, medium. | Probe sonication for 60 s at 30 % amplitude and 20 kHz.  Cooling system used. | V79  (hamster)  Duration of exposure = 24 or 48 h  Concn.  1-100 mg/mL | 7 | Cell viability was reduced in both a concentration- and time-dependent manner by NM exposure.  Lower genotoxic effect of larger NM agglomerates reported compared to smaller agglomerates. |
| Dai et al., 2019 (72) | TiO_2_ primary NM size = 30 or 100 nm.  Size characterisation provided by suppliers. | NM concentration = 5 mg/mL PBS medium.  Volume not reported. | US bath sonication for 30 min.  Temperature not reported. Suspensions then diluted with DMEM. | RAW 264.7 (mouse)  Duration of exposure = 24 h  Concn.  50 -400 µg/mL | 5 | Cell viability was reduced in both a concentration- and time- dependent manner by NM exposure.  Phagocytic rate was decreased in a dose-dependent manner to NM exposure.  Beclin-1 (autophagy regulator) was upregulated in a dose-dependent manner by NM exposure. |
| Murugadoss et al., 2020 (16) | TiO_2_ primary NM size = 17 and 117 nm.  Size characterisation provided by suppliers. | NM concentration not reported.  Cell culture medium.  Volume not reported. | Dispersed at different pH conditions. Followed by probe sonication, delivering 7056 J/ Time and temperature not reported. Suspensions then stabilized with the addition of 0.25 % BSA. pH adjustments to pH 7-7.5 then performed using 0.1M NaOH. | HBE, THP-1 and Caco-2  (human)  Duration of exposure = 24 h  Concn.  4 – 256 µg/mL | 4 | For 17 nm particles: larger aggregates induced a stronger response for glutathione depletion, IL-8 and IL-1β increase, and DNA damage in THP-1 cells compared to smaller aggregates.  For 117 nm particles: No observed differences between larger aggregates and smaller aggregates for each assay exhibited, regardless of cell line. |
| Bettencourt et al., 2020 (65) | TiO_2_  primary NM size = 22, 28 and 30 nm.  Size characterisation provided by suppliers. | NM concentration = 2.56 mg/mL.  Powder prewetted in 0.5 % v/v ethanol (96 %), followed by addition of 0.05 wt% BSA-water.  Volume not reported. | Probe sonication for 16 min.  Cooling system used.  Temperature not reported. | Caco-2, HT29-MTX-E12  (human)  Duration of exposure = 24 h  Concn.  0.14 – 14.3 µg/mL  Simulated digestion of NMs performed prior to addition to their exposure to cells. | 8 | NM agglomerate size for the 22 and 28 nm particles was found to be ~100 nm.  Agglomerate size did not significantly change following the simulated digestion protocol.  NM agglomerate size for 30 nm particles determined to be around 66 nm and decreased to approx. 49 nm following digestion.  After 24 h of exposure of NM to Caco-2 cells, none of the undigested or digested NMs led to a significant decrease in cell survival.  After 24 h of exposure of NM to HT29-MTX-E12 cells, only agglomerates of the 30 nm particles resulted in a reduction in cell viability. This result was most pronounced for digested agglomerates. |
| Brooks et al., 2022 (73) | Sepiolite  primary NM size = 62 % of the sepiolite nanofibers are found to have a length  ranging between 100 and 400 nm.  NM size characterised by researchers. | NM concentration = 2 mg/mL.  10 mM Tris−HCl buffer, pH = 7.5 10 mL suspension. | Probe sonication.  Suspension sonicated three times with an  on/off pulse duration of 10 s at different sonication times (10,  20, 60, and 180 s). The procedure was repeated for each sonication time with amplitude settings of 30, 50, and 100 %.  Temperature not reported. | U2OS and RG37  (human)  Duration of exposure (cell viability) = 24 h  Concn  10 μg/mL | 4 | Calorimetric analysis indicates an increase in temperature with sonication time (0 - 180 s). For 30 % amplitude, the increase in temperature was reported to be around 30 - 50 ^o^C. This temperature increased as amplitude increases to 100 %.  Sonication decreased the sizes of NM fibers.  The proportion of nanofibers with lengths less than 100 nm increased from 7 to 10 % and 16 % after sonication treatment of 0, 10, and 60 s, respectively.  Sonication time did not significantly influence the cell viability (for both cell types, using a LIVE/DEAD assay).  Hemolytic activity of human blood cells decreased (80 - 20 %) when sonication time was increased (i.e. from 0 - 180 s). |
| Ferrante et al., 2023 (67) | TiO_2_. E171 primary NM size = 60 nm TiO_2_, not reported for E171.  NM size characterised by researchers. | NM concentration = 1000 mg/L.  Cell culture medium.  Volume not reported. | Sonication at 300 W for 15 min. Probe or US sonication not specified.  Temperature not reported.  Samples sonicated in a polypropylene vial. | Caco-2, HCT-116  (human)  Duration of exposure = either 24 or 72 h.  Concn.  typically, 0.1 - 500 mg/L  Cell viability dose 0.001-1000 mg/L | 3 | Cell viability was reduced in a dose dependent manner in response to NM exposure, with concentrations > 1 mg/L exhibiting the most pronounced decrease.  A significant increase in DNA damage was observed following 72 h of exposure, where NM exposure at concentrations > 100 and 500 mg/L, for HCT116 and Caco-2 cells resulted in a marked increase, respectively.  Expression of Bcl-2 and Bax markers remained similar to the control following NM exposure.  Exposure of cells to the NM resulted in comparable cell proliferation to unexposed cells. |
