# Supplementary material for: Investigating the impact of the dispersion protocol on the physico-chemical identity and toxicity of nanomaterials: a review of the literature with focus on TiO2 particles
Source: Part Fibre Toxicol. 2025 May 13;22:11. doi: 10.1186/s12989-025-00627-8 (PMC12070512; doi:10.1186/s12989-025-00627-8)
Supplement: Supplementary file 4 — Supplementary Material 4: Additional file 4: Table S4. Summary on the reporting of the influence of the storage conditions of NM stock suspensions on NM PC identity and toxicity. Data summarising test material, dispersion methodology, storage temperature including Nano Score and Klimisch Score of research articles which report the influence of the storage conditions of NM stock suspensions on NM PC and toxicity. [file 12989_2025_627_MOESM4_ESM.docx]

**Additional file 4**

**Table S4**: **Summary on the reporting of the influence of the storage conditions of NM stock suspensions on NM PC identity and toxicity.**

| **Study Ref.** | **Test Material** | **Dispersion methodology** | **Storage Temperature  (°C)** | **Model System incl. Exposure Details** | **Nano Score (0-10)** | **Reported Findings** |
| --- | --- | --- | --- | --- | --- | --- |
| **Toxicity of NM not assessed – no Klimisch Score** | | | | | | |
| Bihari et al., 2008 (31) | TiO_2_ primary NM size = 10 x 40 nm  Size characterisation provided by suppliers. | Probe sonication.  NM dispersed in PBS (with and without HSA)  NM concentration 0.02mg/mL.  Volume not reported. | Not reported | N/A: impact on PC identity assessed. | 5 | TiO_2_ (rutile) exhibited an increase in NM particle size from 0 – 24 h. After 24 h, a plateau in NM size was observed.  When human serum albumin (HSA) is added to dispersant media formulation, NM size is stable over 1 week, showing no change. |
| Murdock et al., 2008 (30) | Cu  primary NM size = 40, 60 80 nm  Size characterisation provided by suppliers. | Probe sonication.  NM dispersed in DI H_2_O.  NM concentration = 1 mg/mL.  Volume not reported. | 4 °C, and prior to each measurement, the stock suspensions were removed from the refrigerator, warmed to room temperature, vortexed, and then diluted to 25 µg/mL. | N/A: impact on PC identity assessed. | 5 | Over 34 days, an increase in NM particle size was observed for all Cu NM sizes.  Over similar timescales, the zeta potential was observed to fluctuate.  For Cu 60 nm, the zeta potential continued to decrease in size, while for Cu 40 nm and Cu 80 nm, the zeta potential decreased initially, followed by a sharp increase and began to plateau from day 10 onwards. |
| **Klimisch Score = 1** | | | | | | |
| no studies identified | | | | | | |
| **Klimisch Score = 2** | | | | | | |
| no studies identified | | | | | | |
| **Klimisch Score = 3** | | | | | | |
| Kittler et al., 2010 (85) | Ag (poly(vinylpyrrolidone)-stabilized)  primary NM size = 50 ± 20 nm  NM size characterised by researchers. | No sonication used.  NM dispersed in ultrapure water.  NM stock concentration = 1 mg/mL.  50 µL of Ag NM added 1mL of cell culture medium. | 5 °C. After 3 days, 1 month, and 6 months, respectively, particles were taken for hazard studies. | MSCs  (human)  Duration of exposure = 24 h  Concn.  50, 25, 20, 15, 5, 2.5, and 1 mg/mL | 4 | Aged silver nanoparticles, i.e., after 1 month and 6 months of immersion, caused complete cell death at concentrations ≥ 5 µg/mL.  A reduction in cell viability to 70 % was observed for Ag NMs aged for 3 days, at concentrations ≥ 25 µg/mL. |
